# Machine-learning framework for conditional estimation and scenario-based projection of the heat index for public health interventions

**DOI:** 10.1371/journal.pone.0348202

**Published:** 2026-04-28

**Authors:** Mohammad A. H. Mollah, Amrin Binte Ahmed, Zihanul Islam Zijan, Ananta Asim Joy, Rumana Rois

**Affiliations:** 1 Department of Natural Resources and Environmental Design, North Carolina Agricultural and Technical State University, Greensboro, North Carolina, United States of America; 2 Department of Statistics and Data Science, Jahangirnagar University, Dhaka, Bangladesh; 3 Department of Geography and Environmental Studies, University of Rajshahi, Rajshahi, Bangladesh; 4 Department of Mathematical & Physical Sciences, East West University, Dhaka, Bangladesh; 5 Department of Statistics and Data Science, Jahangirnagar University, Dhaka, Bangladesh; The Chinese University of Hong Kong, HONG KONG

## Abstract

**Background:**

Heatwaves are becoming more frequent and intense in Bangladesh, particularly in urban areas such as Dhaka, where the combined effects of extreme heat and humidity pose serious public health risks. Temperature-based warning systems frequently underestimate physiological heat stress.

**Methods:**

This study proposes a machine-learning framework to estimate and project the Heat Index (HI) using 3-hourly meteorological records from 2014 to 2023. Because HI is a deterministic function of air temperature and relative humidity, the framework performs conditional estimation of HI based on meteorological predictors rather than independently forecasting HI itself. Five models: ARIMAX, SARIMAX, Random Forest Regressor (RFR), XGBoost, and Long Short-Term Memory (LSTM), were trained using air temperature, relative humidity, and seasonal indicators as predictors. For scenario-based projections beyond 2023, future temperature and humidity were approximated using historical monthly averages, generating scenario-based HI projections that preserve seasonal and diurnal patterns. These projections represent climatological scenarios rather than true meteorological forecasts.

**Results:**

The Random Forest Regressor (RFR) achieved the highest conditional estimation accuracy, with the lowest RMSE (0.85°C) and highest R² (0.987) on the test set. Empirical 95% prediction intervals achieved 98.85% coverage, indicating slightly conservative uncertainty bounds. Scenario-based projections yielded mean HI values of 29.02°C (optimistic), 29.90°C (moderate), and 31.33°C (pessimistic). A substantial proportion of projected 3-hourly periods fall within the “Extreme Caution” category (32–41°C), indicating persistently elevated heat-stress exposure under climatological assumptions.

**Conclusion:**

The proposed framework demonstrates strong potential for generating high-resolution scenario-based HI projections by capturing nonlinear temporal dynamics and sub-daily variability. These findings can support scenario-based early warning systems and inform adaptive urban heat-management strategies in climate-vulnerable cities such as Dhaka, although results should be interpreted as conditional projections rather than deterministic forecasts. Unlike conventional HI studies, this framework translates meteorological inputs into high-resolution, operational heat-risk insights by modeling temporal persistence at sub-daily scales.

## 1. Introduction

The increasing frequency and severity of extreme weather events, particularly heatwaves, are among the most pressing consequences of climate change. A heatwave is generally defined as a prolonged period of excessively high temperatures, often accompanied by elevated humidity; however, definitions vary regionally depending on the local climate, population vulnerability, and socioeconomic conditions [1,2]. In South Asia, including Bangladesh, heatwaves have become a major public health concern. In densely populated urban areas, inadequate housing, limited green space, and unplanned urbanization further intensify thermal stress [3,4]. Dhaka, the capital of Bangladesh, is among the world’s most rapidly urbanizing and heat-vulnerable megacities, where the urban heat island effect and socioeconomic disparities exacerbate exposure and risk [5].

Global climate change has intensified the risk of heatwaves. Between 1901 and 2001, global temperatures increased by approximately 0.6 °C, followed by an additional 1–2 °C rise since the 1980s, contributing to a three-fold increase in global heatwave days over the past four decades [6,7]. Evidence from cities such as Detroit (USA) and Thessaloniki (Greece) highlights the severe health impacts of extreme heat and the importance of robust Heat Health Warning Systems (HHWSs) [8,9]. South Asia, Europe, and Eastern North America are among the regions most affected by heatwave-related mortality [10], and older adults are particularly vulnerable, experiencing up to five times more heatwave days than the global average [11]. Previous studies suggest that heatwave duration could increase substantially by the 2050s, with new hotspots emerging across Central Africa, Northern South America, and Southeast Asia [12]. Furthermore, the frequency of extreme wet-bulb temperature events may increase by 100–250 fold by 2080, potentially exposing hundreds of millions of people to deadly heat conditions [13]. Despite these escalating risks, most research and forecasting efforts remain concentrated in high-income, mid-latitude countries, leaving highly exposed, low-resource regions, such as Bangladesh, underrepresented [14].

Bangladesh has experienced a notable increase in the frequency and intensity of heatwaves, posing a threat to public health, agriculture, and economic stability [15]. These events are driven by solar insolation, temperature advection, and moisture incursion, with the most intense heatwaves occurring in the southwest and central-western regions [16]. Vulnerable groups, such as the Santal community in Rajshahi, face disproportionate impacts due to disrupted food systems and increased heat-related illnesses [17]. The 2024 heatwave, with temperatures reaching 42 °C (approximately 6 °C above the long-term average), was among the most severe since records began in 1948 [18,19], resulting in widespread school closures and at least 15 heatstroke-related deaths [18,20]. These events highlight the need for proactive adaptation measures, including early warning systems, risk communication, and targeted cooling interventions [15].

Current early warning systems in Bangladesh primarily rely on air temperature to define and predict extreme heat. However, temperature alone does not reflect human thermal discomfort in humid-subtropical climates [21]. The Heat Index (HI), which combines air temperature and relative humidity, provides a more meaningful measure of perceived heat and physiological strain [22]. High humidity impairs evaporative cooling, meaning that two days with the same temperature can present vastly different health risks depending on moisture levels [23]. Therefore, incorporating HI into heat-risk assessment frameworks can strengthen early warnings by aligning alerts with actual physiological dangers rather than temperature thresholds alone.

Recent studies have demonstrated that HI-based modeling approaches provide improved performance over temperature-only methods in predicting heatwave intensity and health impacts [24–26]. For example, Yodpibul et al. (2021) developed an autocorrelated decomposition model for HI in Thailand that exceeded the benchmark performance [24], whereas Lateef et al. (2021) and Uppal et al. (2021) employed deep learning and bias-adjustment techniques to improve HI predictions across diverse climates [25,26]. Statistical post-processing has further enhanced the accuracy of short-term HI ensemble forecasts [27]. However, most existing research focuses on mid-latitude or high-income countries, limiting its applicability to low-resource tropical cities, such as Dhaka.

To address this gap, the present study developed a machine-learning–based framework to conditionally estimate and project the Heat Index (HI) in Dhaka using 3-hourly meteorological observations from 2014 to 2023. Because HI is deterministically derived from air temperature and relative humidity, the modeling task is framed as conditional HI estimation based on meteorological predictors rather than independent HI forecasting. This framework integrates temperature, relative humidity, and seasonal indicators to capture the dynamic drivers of thermal stress. Five models: ARIMAX, SARIMAX, Random Forest Regressor, XGBoost, and Long Short-Term Memory (LSTM), were evaluated to compare conditional estimation performance across statistical, ensemble, and deep learning approaches, with model accuracy assessed using multiple evaluation metrics. For the extended projection period (2024–2027), future temperature and humidity were approximated using historical monthly averages to create scenario-based predictor inputs. These projections represent climatological scenarios based on historical seasonal patterns rather than true meteorological forecasts and are intended to explore plausible future heat-stress conditions under stationary assumptions. By linking scenario-based projections to operational intervention thresholds, this study provides high-resolution insights for early warning systems and urban heat adaptation planning, offering a scalable tool for enhancing climate resilience in one of the world’s most heat-vulnerable megacities. Although the Heat Index is a deterministic function of temperature and relative humidity, the key contribution of this study lies in modeling its nonlinear temporal persistence, sub-daily variability, and conditional dynamics using machine-learning approaches. This enables the transformation of meteorological inputs into high-resolution, operationally relevant heat-risk information that extends beyond the static HI formulation used in conventional studies.

## 2. Materials and methods

### 2.1. Study area and data source

This study examined heat stress conditions in Dhaka, the capital of Bangladesh and one of the most densely populated megacities in South Asia. Dhaka experiences persistent thermal stress owing to high temperatures, elevated humidity, and pronounced urban heat island effects. Meteorological data were obtained from the Climate Division of the Bangladesh Meteorological Department (BMD) from January 1, 2014, to December 31, 2023. As the national climate authority, the BMD provides standardized and quality-controlled meteorological observations. Dry-bulb temperature (°C) and relative humidity (%) were recorded at three-hour intervals following synoptic hours (00:00, 03:00, 06:00, 09:00, 12:00, 15:00, 18:00, and 21:00 UTC). These variables constitute the primary inputs for the Heat Index (HI) estimation. The data were supplied in a wide format with temporal identifiers (year, month, day, and hour), allowing for systematic temporal alignment and time-series analysis. The use of three-hourly observations enables the modeling framework to capture intra-day variability in heat stress, including peak exposure periods and limited nocturnal cooling, which are often obscured in daily or coarser temporal aggregations. Such a high-frequency resolution is particularly relevant for HI-based health risk assessments, in which cumulative short-term exposure and diurnal persistence of thermal stress play critical roles.

### 2.2. Data preprocessing and validation

The dataset consisted of three-hourly observations of dry-bulb temperature and relative humidity for Dhaka from January 1, 2014, to December 31, 2023, with eight daily measurements. A rigorous preprocessing protocol was applied to ensure data integrity and methodological transparency. The raw temperature and humidity files were first transformed from a wide to a long format, creating a single record for each timestamp. The two datasets were then merged through multi-key matching using year, month, day, and hour information, after which a standardized date–time index was generated. This index was examined to confirm its continuity over the entire 10-year period. Quality control checks were conducted to verify dataset reliability. The merged dataset was examined for missing values at both the timestamp and variable levels, and no missing observations were detected. All dates were validated using strict date-parsing functions to ensure that no invalid dates (such as February 30) were included. Potential outliers were identified using the interquartile range method and cross-checked against raw BMD logs. All extreme values were confirmed to be genuine atmospheric conditions rather than measurement errors; therefore, no values were excluded. To prepare the dataset for modeling, a seasonal variable (winter, spring, summer, and autumn) was created based on the month values. The predictors used in the machine learning algorithms (temperature, relative humidity, seasonal indicators, and temporal variables) were normalized using min-max scaling, whereas the statistical models were fitted using the raw values. Multicollinearity among exogenous variables was assessed using Variance Inflation Factors (VIF), with all VIFs below 5, indicating no concerning degree of linear dependence. After preprocessing, the dataset consisted of 29,216 complete and validated three-hourly observations, ensuring full temporal continuity that was suitable for conditional estimation and scenario-projection analysis.

### 2.3. Feature engineering for heat index (HI)

The Heat Index (HI) was computed to quantify the combined effects of air temperature and relative humidity on perceived thermal stress, serving as an apparent temperature metric widely used in public health and heat warning systems. The HI was calculated following the approach developed by Rothfusz (1990) and adopted by the U.S. National Weather Service (NWS) [28]. The two-stage Heat Index computation procedure is summarized in Table 1.

**Table 1:** Methodology for Computing Heat Index (HI): Two-stage Calculation Using Steadman’s Formula and Rothfusz Regression Model with Empirical Adjustments [28].

**Stage 1: Preliminary Estimate (Steadman’s Formula):**
For all observations, an initial estimate of the HI was computed using the simplified empirical formula based on Steadman’s method:
$HI_{simple}=0.5\times [T+61.0+((T-68.0)\times 12)+(RH\times 0.094)]$ (1)
Where T is the air temperature (°F), and RH is the relative humidity (%). The preliminary HI was obtained by averaging this value with the actual temperature.
**Stage 2: Rothfusz Regression Model:**
For cases where the preliminary HI was ≥ 80°F (26.7°C), the full Rothfusz multiple-regression equation was applied:
$\begin{array}{c} HI=-42.379+\text{2}\mathrm{.0}\text{49}0\text{1523T}+\text{1}0.\text{14333127RH}\\ -0.\text{22475541TRH}-0.00\text{683783}T^{\text{2}}-0.0{\text{5481717RH}}^{\text{2}}\\ +0.00{\text{122874T}}^{\text{2}}\text{RH}+0.000{\text{85282TRH}}^{\text{2}}-0.00000{\text{199T}}^{\text{2}}{\text{RH}}^{\text{2}}\; \end{array}$ (2)
Two empirical adjustments were then applied under specific humidity–temperature conditions:
**• Low Humidity Adjustment** If RH < 13% and 80°F ≤ T ≤ 112°F, a negative adjustment was subtracted:
$ADJ=\left(\frac{13-RH}{\text{4}}\right)\sqrt{\frac{17-\left|T-95\right|}{17}}$ (3)
**• High Humidity Adjustment** If RH > 85% and 80°F ≤ T ≤ 87°F, a positive adjustment was added:
$ADJ=\left(\frac{RH-85}{10}\right)\left(\frac{87-T}{\text{5}}\right)$ (4)

The final HI estimates were then converted back to degrees Celsius to maintain consistency with other climatic variables. Based on the computed HI values, heat stress severity was categorized into five levels following the NWS guidelines: Normal, Caution, Extreme Caution, Danger, and Extreme Danger [29]. Because the Heat Index is deterministically derived from temperature and relative humidity, it does not constitute an independent meteorological variable. Accordingly, the modeling framework estimates HI conditionally based on meteorological predictors rather than independently forecasting HI itself. The classification of Heat Index values and associated health risk levels is presented in Table 2.

**Table 2:** Classification of HI values and associated health risk levels [29].

Category	HI (°C)	Health Implications
Normal	< 27°C	No significant risk under normal conditions.
Caution	27–32°C	Possible fatigue with long exposure or physical activity.
Extreme Caution	32–41°C	Risk of sunstroke, muscle cramps, or heat exhaustion.
Danger	41–54°C	High risk of heat exhaustion or sunstroke; medical attention may be needed.
Extreme Danger	≥ 54°C	Very high risk of heatstroke; likely to be life-threatening without quick action.

### 2.4. Dataset structure

The final dataset comprised 29,216 complete three-hourly records spanning 2014–2023, each containing temperature, relative humidity, HI, HI category, season, and a standardized date–time index. No missing values were found. A significant negative correlation was observed between temperature and relative humidity (r = –0.356, p < 0.001), consistent with the known meteorological patterns. Seasonal analysis showed the expected climatological variation, with mean summer temperatures of 29.5°C (SD = 2.35) and winter averages of 20.7°C (SD = 4.21). HI categories indicated heightened thermal stress during summer months, with 11.24% of observations falling under “Danger” or “Extreme Danger.” To prevent data leakage, records from 2014 to 2021 were used for training, while 2022–2023 were reserved for testing. This chronological split ensured that no future information influenced the model training. All lagged HI features were generated using strictly past observations within each temporal partition, ensuring that no information from the test period was used during feature construction.

### 2.5. Conditional estimation and projection models

To conditionally estimate and generate scenario-based projections of Heat Index dynamics in Dhaka, we employed a set of statistical and machine-learning models. All models incorporated exogenous variables, including air temperature, relative humidity, seasonal indicators, and temporal features, to enhance their estimation performance. The selected models included Autoregressive Integrated Moving Average with Exogenous Inputs (ARIMAX), Seasonal ARIMAX (SARIMAX), Long Short-Term Memory networks (LSTM), Random Forest Regression (RFR), and Extreme Gradient Boosting (XGBoost). Collectively, these models capture both linear and nonlinear dependencies, as well as the influence of meteorological drivers on HI variability.

The selection of these five models was guided by the need to evaluate the conditional estimation performance across distinct methodological paradigms under high-frequency climatic conditions. ARIMAX and SARIMAX represent classical statistical approaches capable of modeling linear dependence and seasonal structure in time-series data, providing a transparent benchmark. In contrast, RFR and XGBoost are ensemble-based machine learning models designed to capture nonlinear interactions between temperature, humidity, and temporal features that are characteristic of heat stress dynamics. The LSTM model was included to assess the ability of deep learning architectures to learn sequential dependencies in sub-daily climate data. This comparative framework enables a balanced assessment of the model interpretability, predictive accuracy, and generalization performance across different modeling approaches. All projections are generated through iterative 3-hour-ahead recursive estimation, producing multi-year scenario trajectories rather than direct long-horizon forecasts.

#### 2.5.1. ARIMAX model.

The ARIMAX model extends the classical ARIMA framework by incorporating external predictor variables, known as exogenous variables, to improve forecasting accuracy [30]. Like ARIMA, the ARIMAX model accounts for autoregressive (AR), integrated (I), and moving average (MA) components, but it additionally models the influence of external covariates on the target time series [30,31]. Mathematically, the ARIMAX model is represented as [32]:


$$\varphi \left(L\right)(1-L{)}^{d}Y_{t}=\theta (L)X_{t}+\theta (L)Z_{t},where\{Z_{t}\}\sim WN(0,\sigma^{\text{2}})$$ (5)

Here L is the lag operator; φ(L) and θ(L) are polynomials of order p and q, respectively; and $Z_{t}$ is a white noise error term. The inclusion of exogenous variables allows the model to capture additional information from external factors, improving forecast precision, especially in complex environmental and meteorological time series [33].

#### 2.5.2. SARIMAX model.

The SARIMAX model extends the SARIMA framework by incorporating exogenous variables (X), which serve as external inputs to improve model accuracy. These additional features help reduce prediction errors, address autocorrelation issues, and enhance overall forecasting performance [34]. A SARIMAX model is denoted as $SARIMAX\left(p,d,q\right)(P,D,Q{)}_{s}$, where (P, D, Q) are the seasonal counterparts of the non-seasonal ARIMA parameters and s denotes the seasonal period (e.g., s = 8 for three-hourly data over a daily cycle) [35–37]. The SARIMAX model is written as [36]:


$$\varphi \left(L\right)\varphi \left(L^{s}\right)(1-L{)}^{d}(1-L^{s}{)}^{D}Y_{t}=\alpha_{k}x_{k,t}+\theta \left(L\right)\theta \left(L^{s}\right)Z_{t},where\left\{Z_{t}\right\}\sim \;WN\left(0,\sigma^{\text{2}}\right)$$ (6)

In this formulation, $\varphi \left(L^{s}\right)$ and $\theta \left(L^{s}\right)$ are polynomials of seasonal autoregressive and seasonal moving average terms, respectively, while $(1-L^{s}{)}^{D}$ introduces seasonal differencing. The term $x_{k,t}$ refers to the kth exogenous factors at time t and $\alpha_{k}$ represents its corresponding coefficient. This formulation enables SARIMAX to capture both short-term and seasonal dependencies while integrating temperature, humidity, and other meteorological variables that influence HI. The model is widely used in climate and environmental forecasting due to its ability to incorporate domain-specific information [38].

#### 2.5.3. LSTM model.

The Long Short-Term Memory (LSTM) network is a specialized form of Recurrent Neural Network (RNN) designed to overcome the limitations of learning long-range temporal dependencies, particularly vanishing and exploding gradients [39]. LSTM networks use a memory cell regulated by three gating mechanisms: input, forget, and output gates that control the flow of information. The LSTM cell is governed by the following equations:


$$f_{t}=\sigma (W_{f}\cdot [h_{t-1},X_{t}]+b_{f}),$$ (7)

$$i_{t}=\sigma (W_{i}\cdot [h_{t-1},X_{t}]+b_{i}),$$ (8)

$${\hat{C}}_{t}=tanh(W_{c}\cdot [h_{t-1},X_{t}]+b_{c}),$$ (9)

$$C_{t}=i_{t}\cdot {\hat{C}}_{t}+f_{t}\cdot C_{t-1},$$ (10)

$$o_{t}=\sigma (W_{o}\cdot [h_{t-1},X_{t}]+b_{o}),$$ (11)

$$h_{t}=o_{t}\cdot tanh\left(C_{t}\right)$$ (12)

Here, σ represents the sigmoid activation function, tanh is the hyperbolic tangent function,  . denotes element-wise multiplication, $X_{t}$ is the input at time t, $h_{t}$ is the hidden state, and $C_{t}$ is the cell state. The parameters W and b correspond to weight matrices and bias vectors learned during training [40,41].

#### 2.5.4. RFR model.

Random Forest Regression (RFR) is an ensemble learning technique that constructs multiple decision trees using bootstrapped samples of the training data and aggregates their outputs to improve predictive performance [42]. At each split, a random subset of features is selected, reducing correlation among trees and enhancing generalization. The final prediction is the average of individual tree predictions:


$${\hat{f}}_{rf}\left(x\right)=\frac{\text{1}}{K}\sum_{k=1}^{K}T(x;\theta_{k})$$ (13)

where $T(x;\theta_{k})$ denotes the prediction of kth tree trained under random vector $\theta_{k}$ and K is the total number of trees. RFR effectively handles high-dimensional data, reduces overfitting, and captures complex nonlinear relationships, making it suitable for modeling nonlinear heat-stress dynamics in environmental time-series data [43].

#### 2.5.5. XGBoost model.

XGBoost is an optimized gradient boosting algorithm that employs a regularized objective function to improve model generalization and computational efficiency [44]. It constructs trees sequentially by minimizing a second-order Taylor-expanded loss function:


$$L\left(\phi \right)\approx \sum_{i}[g_{i}\phi \left(x_{i}\right)+\frac{\text{1}}{\text{2}}h_{i}\phi^{\text{2}}\left(x_{i}\right)]+\Omega (\phi )$$ (14)

where $g_{i}$ and $h_{i}$ are the first and second derivatives of the loss function, and Ω(ϕ) is a regularization term controlling model complexity. The overall objective function is defined as:


$$obj\left(\theta \right)=\sum_{i}l(y_{i},{\hat{y}}_{i})+\sum_{k}\Omega (f_{k})$$ (15)

where $l\left(y_{i},{\hat{y}}_{i}\right)$ represents the training loss and $f_{k}$ are the individual trees. XGBoost’s scalability, regularization, and high predictive accuracy make it well-suited for climate-related time-series modeling tasks [45].

#### 2.5.6. Preparation of future predictor variables.

To generate scenario-based Heat Index (HI) projections for the period 2024–2027, a synthetic 3-hourly future time grid was constructed spanning from 1 January 2024 to 31 December 2027 at 3-hour intervals, consistent with the historical temporal resolution. For each future timestamp, calendar-based predictors (hour, day, month, and season) were derived, and seasonal indicators were encoded using one-hot encoding consistent with the training dataset. Future temperature and relative humidity values were initialized using historical monthly climatological averages computed from the 2014–2023 dataset. These monthly means were expanded to 3-hourly resolution to preserve seasonal structure. Three alternative meteorological scenarios were then constructed by applying deterministic mean shifts combined with scenario-specific stochastic variability to the baseline climatology. In the optimistic scenario, temperature was decreased by 0.5°C and relative humidity by 2 percentage points, with low Gaussian noise added to represent limited short-term variability. The moderate scenario retained the historical monthly means without systematic shifts but incorporated moderate stochastic variability to reflect typical intra-seasonal dispersion. In the pessimistic scenario, temperature was increased by 1.0°C and relative humidity by 3 percentage points, with elevated Gaussian noise to represent greater variability and amplified heat stress conditions. Stochastic perturbations were generated using a fixed random seed to ensure reproducibility across simulations. HI projections were generated recursively using a 3-hour-ahead conditional estimation framework. Lagged HI predictors (1-step, 3-step, and 6-step lags) were dynamically updated at each recursive step using previously predicted values. For the initial projection steps, the final six observed HI values from 2023 were used to initialize lag features. These projections should not be interpreted as deterministic climate forecasts but rather conditional scenario analyses designed to assess potential heat-stress dynamics under alternative meteorological assumptions. The effective forecasting horizon of the framework is short-term (3-hour ahead recursive estimation), while longer-term outputs (2024–2027) represent scenario-based projections rather than true forecasts.

#### 2.5.7. Empirical tree-based prediction intervals.

For the Random Forest Regressor (RFR), predictive uncertainty was quantified using empirical tree-level dispersion. At each prediction step, individual tree predictions from all ensemble members were retained. The mean prediction was calculated as the average across trees, and 95% empirical prediction intervals were constructed using the 2.5th and 97.5th percentiles of the tree-level prediction distribution:


$$\begin{array}{c} \hat{y_{t}}=\frac{\text{1}}{B}\sum_{b=\text{1}}^{B}T_{b}\left(X_{t}\right) \end{array}$$ (16)

$$\begin{array}{c} {PI}_{\text{9}\text{5}\%}=\left[Q_{\text{2}.\text{5}}\left(T_{b}\left(X_{t}\right)\right),\;Q_{\text{9}\text{7}.\text{5}}\left(T_{b}\left(X_{t}\right)\right)\right] \end{array}$$ (17)

where $T_{b}$ denotes the prediction from the b-th tree and B is the total number of trees.

This non-parametric approach leverages ensemble diversity to approximate conditional predictive dispersion without assuming Gaussian residuals. It is important to note that these prediction intervals reflect model-based conditional uncertainty under fixed meteorological inputs and do not capture the full uncertainty associated with future climate variability or extreme events.

### 2.6. Model validation and robustness assessment

Machine-learning and deep-learning models were evaluated using expanding-window five-fold time-series cross-validation, preserving chronological order to prevent information leakage. Hyperparameters were optimized via grid search within the training folds, and early stopping was applied where appropriate to reduce overfitting. Statistical models (ARIMAX and SARIMAX) were evaluated using residual diagnostics, including autocorrelation analysis, Durbin–Watson statistics for serial correlation, Breusch–Pagan tests for heteroscedasticity, and Q–Q plots for residual normality. No major violations of standard modeling assumptions were detected. Robustness was assessed by examining cross-validation variability, with RMSE showing low dispersion across folds (mean RMSE = 0.89 ± 0.04), indicating stable generalization performance.

For the Random Forest model, predictive uncertainty was evaluated on the hold-out test set using empirical tree-based prediction intervals. The 95% empirical intervals achieved a coverage probability of 98.85%, slightly exceeding the nominal 95% level, indicating conservative but reliable uncertainty quantification. The average interval width was 3.94°C, reflecting moderate short-term predictive dispersion relative to the observed HI range. These uncertainty estimates characterize conditional model dispersion under observed meteorological inputs and should not be interpreted as full probabilistic climate forecasts. These validation procedures help ensure that model performance reflects true generalization ability under temporally ordered data, minimizing the risk of overfitting and information leakage.

### 2.7. Evaluation metrics

To evaluate the conditional estimation performance of the five modeling approaches (ARIMAX, SARIMAX, XGBoost, LSTM, and RFR) for HI estimation in Dhaka, we used multiple statistical metrics. These included Mean Squared Error (MSE), Root Mean Squared Error (RMSE), Mean Absolute Error (MAE), Mean Absolute Percentage Error (MAPE), Mean Absolute Scaled Error (MASE), and the Coefficient of Determination (R2). These metrics collectively assess the accuracy, precision, and explanatory power of each model.


$$\text{MSE}=\frac{\text{1}}{n}\sum_{i=1}^{n}{\left(y_{i}-{\hat{y}}_{i}\right)}^{\text{2}}$$ (18)

$$\text{RMSE}=\sqrt{\frac{\text{1}}{n}\sum_{i=1}^{n}{\left(y_{i}-{\hat{y}}_{i}\right)}^{\text{2}}}$$ (19)

$$\text{MAE}=\frac{\text{1}}{n}\sum_{i=1}^{n}\left|y_{i}-{\hat{y}}_{i}\right|$$ (20)

$$\text{MAPE}=\frac{\text{1}}{n}\sum_{i=1}^{n}\frac{\left|y_{i}-{\hat{y}}_{i}\right|}{y_{i}}\times 100\%$$ (21)

$$\text{MASE}=\frac{\text{1}}{n}\sum_{i=1}^{n}\left(\frac{\left|y_{i}-{\hat{y}}_{i}\right|}{\frac{\text{1}}{n-m}\sum_{j=m+1}^{n}\left|y_{j}-y_{j-m}\right|}\right)$$ (22)

$$R^{\text{2}}=1-\frac{\sum_{i=1}^{n}(y_{i}-{\hat{y}}_{i}{)}^{\text{2}}}{\sum_{i=1}^{n}(y_{i}-\overset{―}{y}{)}^{\text{2}}}$$ (23)

where, n represents the number of observations, $y_{i}$ corresponds to the observed Heat Index values, and ${\hat{y}}_{i}$ represents the model-estimated values. MSE, RMSE, and MAE are scale-dependent metrics, with MAE based on absolute errors and MSE and RMSE based on the squared errors. MAPE is a scale-independent metric calculated from the percentage errors, while MASE provides a scale-free measure suitable for comparing performance across different datasets. MASE additionally evaluates model performance relative to a naive persistence benchmark, representing a simple no-change reference forecast, thereby allowing assessment of whether advanced models outperform baseline predictions. The naive persistence benchmark assumes that the Heat Index at time t equals its value at time t − 1 (3-hour lag), representing a simple no-change reference model. The coefficient of determination ($R^{\text{2}}$) quantifies the proportion of variance in the observed HI explained by the model under conditional meteorological inputs, with values ranging from 0 to 1, where higher values indicate stronger explanatory performance.

**Ethical statement:** This study did not require ethical approval, as it is based solely on secondary data that does not involve any human or animal subjects.

## 3. Results

### 3.1. Data description

This study utilizes a high-resolution climatic dataset comprising 3-hourly observations of meteorological variables from Dhaka, Bangladesh, spanning the period from January 1, 2014, to December 31, 2023. The dataset was obtained from the Bangladesh Meteorological Department (BMD) and includes temperature and relative humidity measurements required to compute the HI. Each day is divided into eight 3-hour intervals, capturing the diurnal cycle with sufficient granularity to assess hourly patterns in thermal discomfort. The dataset encompasses a total of 29,216 observations, enabling robust analysis of seasonal trends, long-term variations, and intraday heat stress dynamics across a ten-year period. Data preprocessing steps included quality control checks and temporal standardization to ensure uniform 3-hourly spacing.

**Fig 1:**
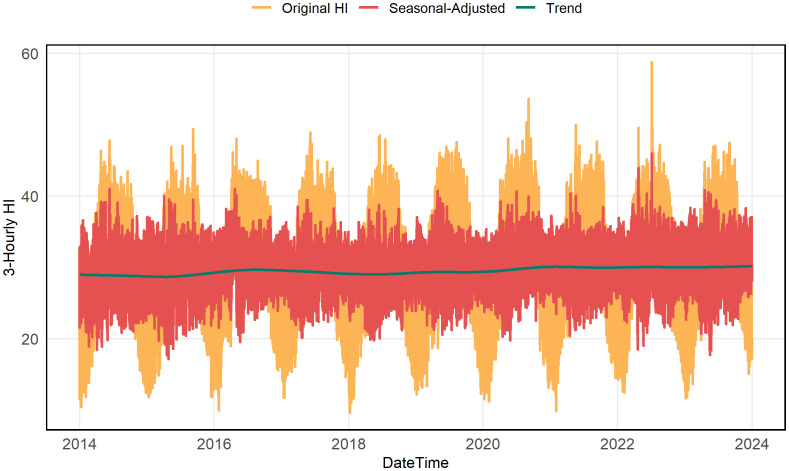
Time series of 3-hourly Heat Index (HI) in Dhaka from 2014 to 2023, decomposed using STL into original values (orange), seasonal-adjusted series (red), and long-term trend (green).

Fig 1 shows the 3-hourly HI series for Dhaka (2014–2023) decomposed using STL into its original, seasonal-adjusted, and trend components. The original HI values display clear annual cycles and notable short-term variability. The seasonal-adjusted series highlights the underlying structure after removing periodic effects, while the trend component reveals a gradual but persistent increase in HI over the decade. This upward trend suggests increasing thermal stress conditions in Dhaka over the study period, as reflected by the long-term HI component. Recognizing these temporal patterns is essential for developing reliable conditional estimation models and scenario-projection frameworks to manage heat-related health risks.

**Fig 2:**
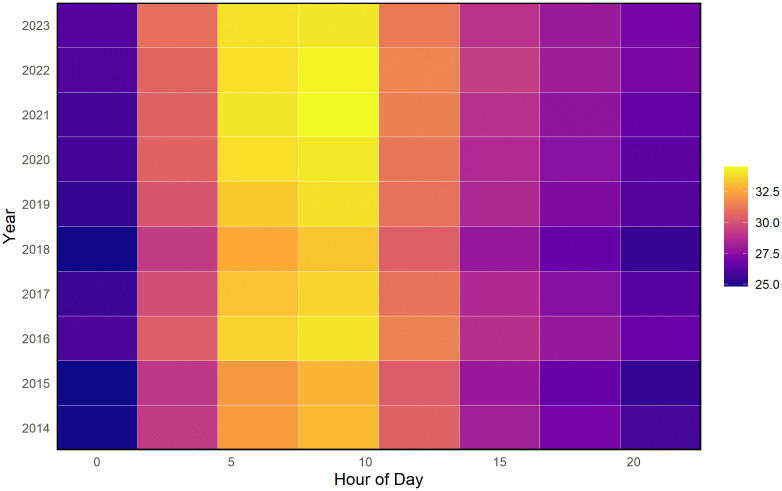
Heatmap of diurnal variation in average HI by year (2014–2023). Colors indicate HI magnitude (°C).

To further understand this heat evolution, Fig 2 shows the diurnal pattern of average HI values from 2014 to 2023. HI remains lowest during early morning and late evening hours and peaks between 06:00 and 12:00, reflecting typical daytime heating driven by rising temperature and humidity. A slight intensification of peak-hour HI after 2019 suggests increasing daytime thermal discomfort, consistent with the long-term upward trend observed in Fig 1. These recurring morning–noon peaks identify time windows associated with heightened heat stress, which are relevant for subsequent risk classification and assessment.

**Fig 3:**
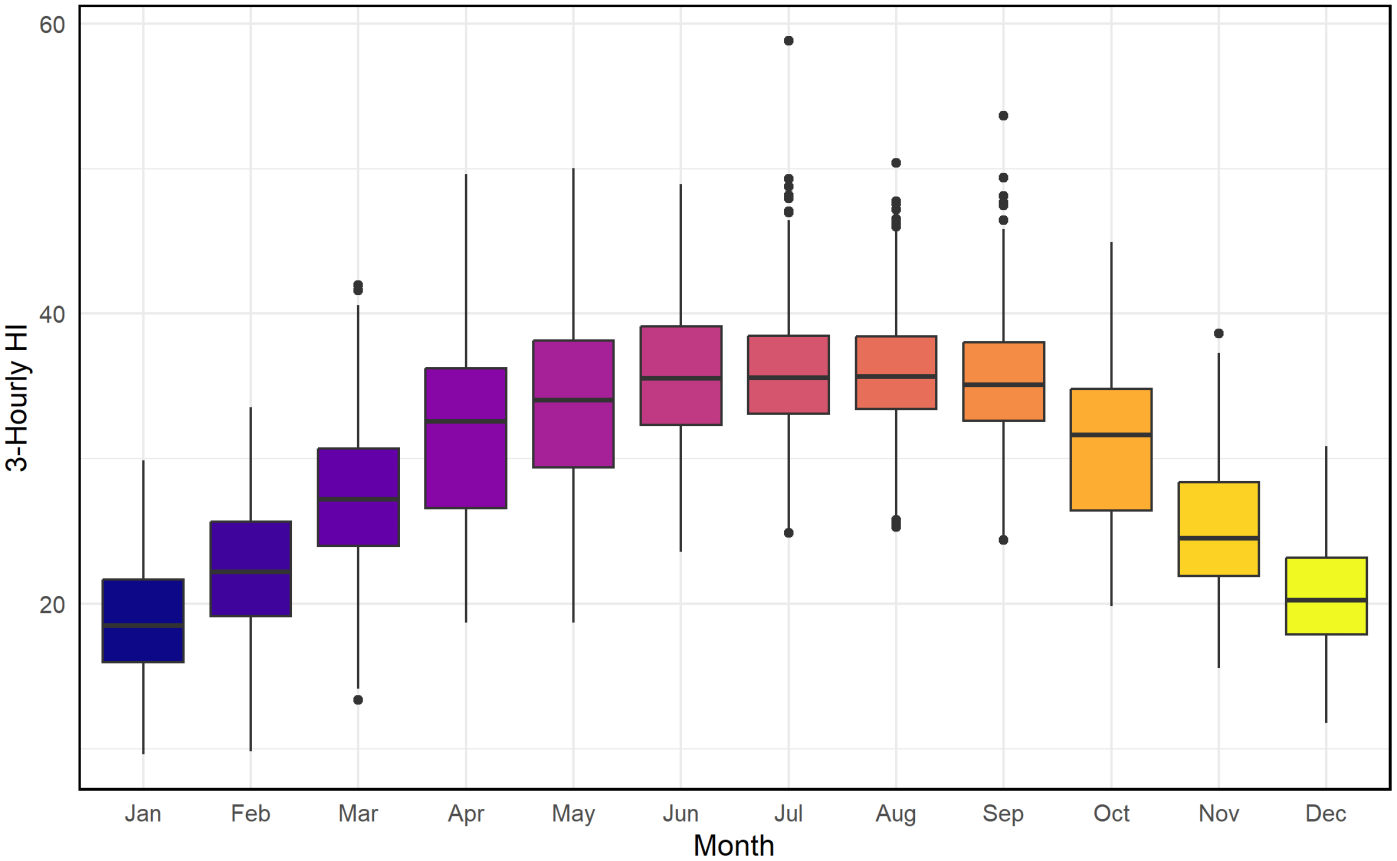
Monthly distribution of 3-hourly HI values (2014–2023) shown as boxplots, indicating median, interquartile range, and extremes.

Fig 3 presents monthly HI distributions over a ten-year period. Winter months (December–February) show low and stable HI values, indicating limited heat stress. From March onward, both median HI and variability increase sharply, reaching their highest levels between June and September, when frequent extreme values (HI > 50°C) occur due to elevated temperature and humidity. HI declines gradually in October–November. These results show that the pre-monsoon and monsoon seasons exhibit the highest HI levels and variability, consistent with commonly applied heat-stress thresholds.

**Fig 4:**
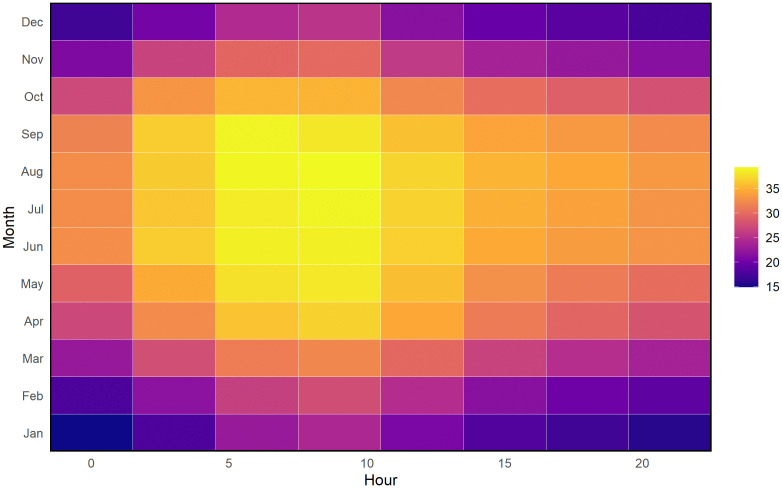
Hourly–monthly heatmap of mean HI values (2014–2023). Each cell represents the average HI for a given hour and month.

Fig 4 illustrates hourly and seasonal patterns of HI across the study period. HI values remain low during January–February, especially at night and early morning (00:00–06:00). As temperatures rise, HI increases sharply from May to September, with the highest levels between 09:00 and 18:00. Bright yellow zones marking HI > 35°C indicate periods of intense heat stress during summer and monsoon afternoons. Elevated nighttime HI during these months reflects limited nocturnal cooling, contributing to persistent thermal discomfort. This combined seasonal–diurnal pattern highlights specific hours and months during which HI reaches its highest levels, providing a detailed temporal context for heat-stress assessment.

**Fig 5:**
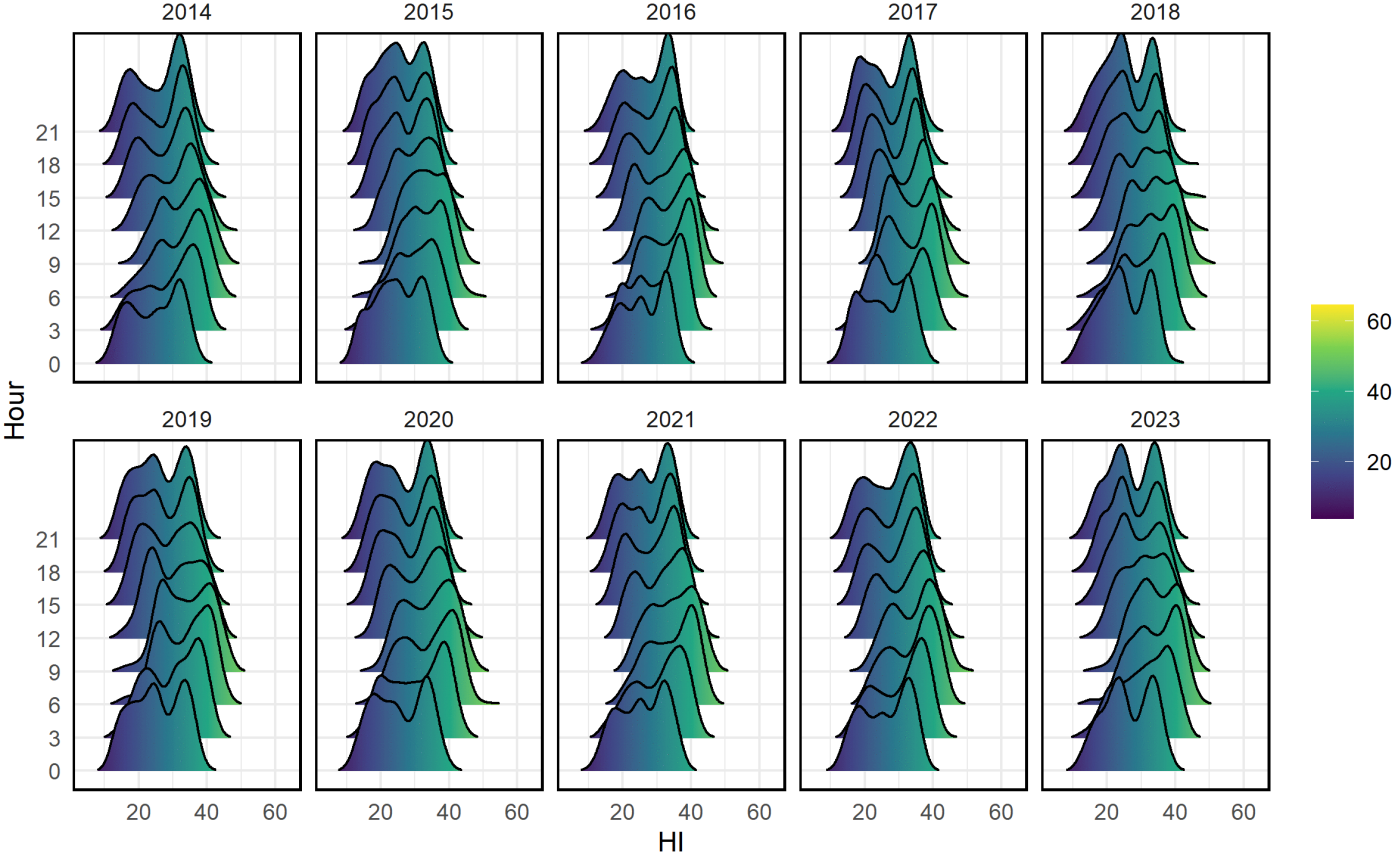
Ridgeline density plots of 3-hourly HI distributions by hour for each year (2014–2023).

These patterns are reinforced in Fig 5, which presents ridgeline density plots of hourly HI distributions for each year. A clear and consistent diurnal cycle emerges: HI is lowest during early morning and late evening hours and peaks between 12:00 and 15:00. In years such as 2019, 2020, and 2023, midday distributions shift rightward, indicating more frequent extreme heat events. The widening and elevation of these curves over time reflect increasing thermal stress severity. This growing intensity of midday heat emphasizes the increasing concentration of high-HI values during specific daytime hours, which is relevant for fine-scale temporal analysis.

**Fig 6:**
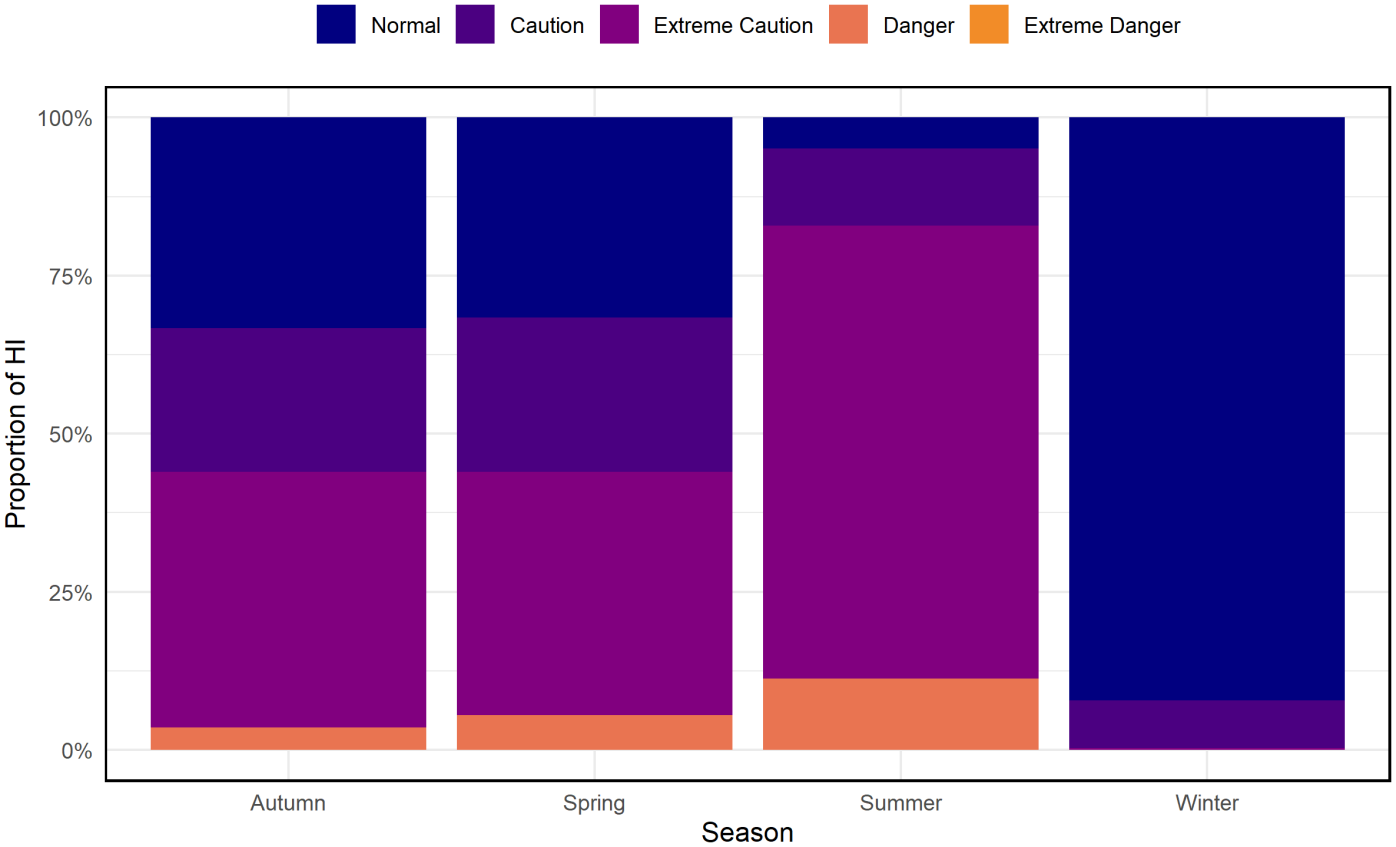
Seasonal distribution of HI risk categories (Normal, Caution, Extreme Caution, Danger, Extreme Danger) for 2014–2023.

To classify the severity of heat stress, Fig 6 shows the seasonal distribution of HI risk categories. Summer has the highest proportion of Extreme Caution and Danger levels, with occasional occurrences of Extreme Danger, indicating substantial heat-related risk. Spring and autumn display mixed conditions dominated by Caution and Extreme Caution levels, while winter is characterized almost entirely by Normal HI, indicating minimal heat stress. These seasonal variations demonstrate that summer exhibits the highest proportion of high-risk HI categories, including Extreme Caution and Danger levels.

**Fig 7:**
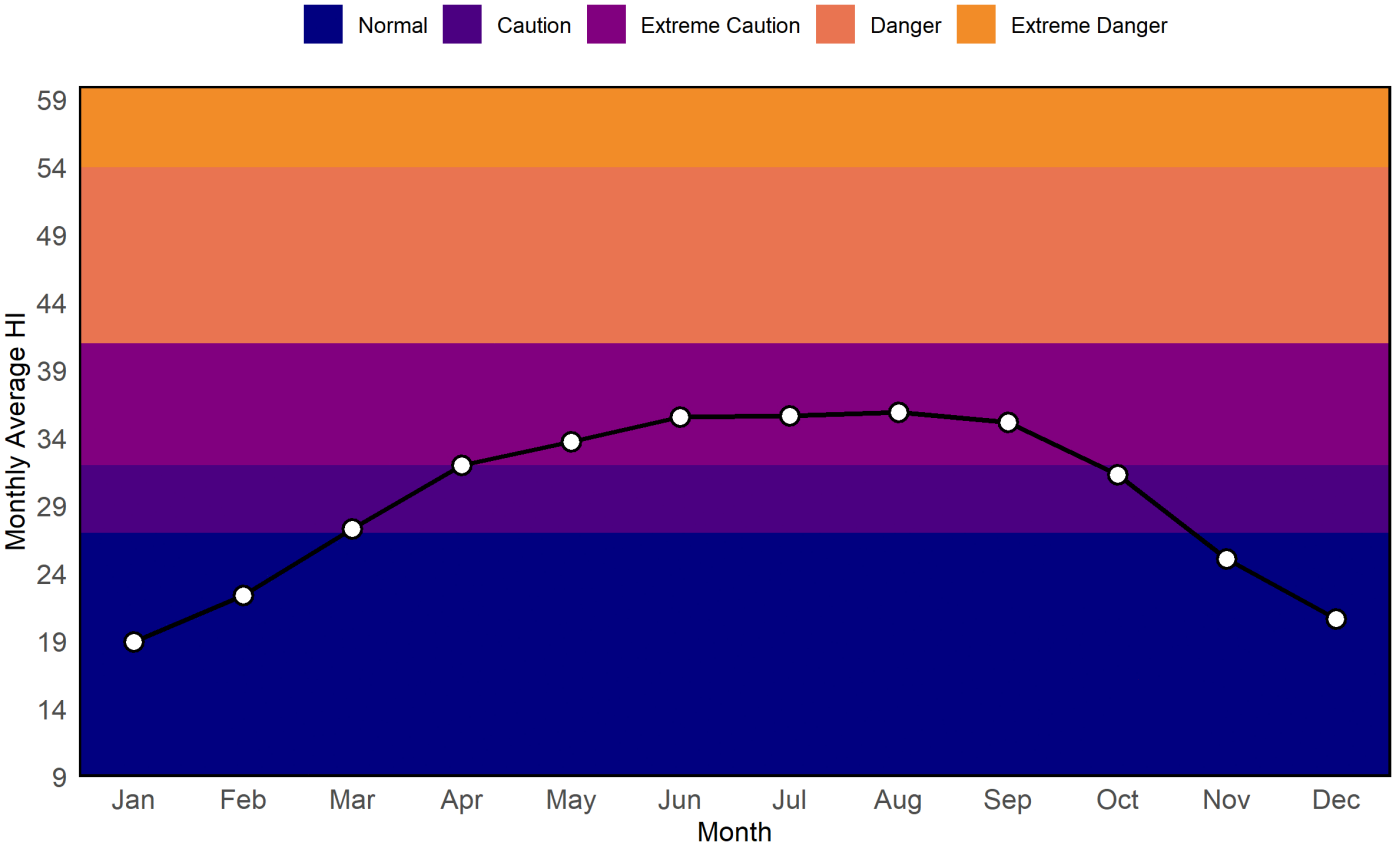
Monthly mean HI values plotted against HI risk categories (2014–2023).

Finally, Fig 7 summarizes monthly mean HI values across risk categories. Winter months (January, February, December) remain within the Normal range, while March enters the Caution zone. From April to October, monthly averages fall consistently in the Extreme Caution category, with peak values in June–August exceeding 35°C. Although monthly means rarely reach the Danger category, the sustained high averages reflect prolonged periods of moderate to high heat stress. This pattern reflects sustained elevated HI conditions during the pre-monsoon and monsoon months, indicating prolonged exposure to moderate-to-high heat stress.

### 3.2. Conditional estimation performance

Table 3 presents a comparative summary of five statistical and machine-learning models applied to conditionally estimate 3-hourly Heat Index (HI) values based on meteorological predictors using multiple evaluation metrics, including MSE, RMSE, MAE, MAPE, MASE, and R². In addition to statistical and machine-learning approaches, model performance was interpreted relative to a naive persistence benchmark (reflected through the MASE metric), which represents a simple baseline assuming no change from the previous observation. Among all models, the Random Forest Regressor (RFR) exhibited the best overall performance, achieving the lowest estimation errors (MSE = 0.7180, RMSE = 0.8473, MAE = 0.5485, MAPE = 1.71%) and the highest coefficient of determination (R² = 0.9872). The high R² reflects the model’s capacity to capture complex temporal dependencies through the inclusion of several lagged HI observations and time-based predictors such as hour of the day, day of the month, month, and season. These features appear to represent short-term autocorrelation and seasonal variability in the HI series. The model was trained and evaluated on a chronologically partitioned dataset, ensuring that no future information was used in training and thereby minimizing data-leakage risk and overfitting. Because HI is derived from temperature and humidity, these results represent conditional estimation accuracy rather than independent forecasting skill.

**Table 3:** Test-period performance comparison across five modeling approaches for 3-hourly HI estimation based on multiple accuracy metrics.

Models	MSE	RMSE	MAE	MAPE	MASE	R^2^
ARIMAX	2.4431	1.5631	1.2034	4.34%	0.5012	0.9565
SARIMAX	4.8727	2.2074	1.6904	5.61%	0.7042	0.9133
RFR	0.7180	0.8473	0.5485	1.71%	0.2285	0.9872
XGBoost	9.1462	3.0243	2.3579	8.27%	0.9821	0.8372
LSTM	4.7841	2.1873	1.4990	5.23%	0.6242	0.9146

To provide a more robust and scale-independent evaluation of conditional estimation accuracy, the Mean Absolute Scaled Error (MASE) was also included. Unlike MAPE, which can appear deceptively small for large-magnitude environmental variables, MASE compares model errors to those of a naive persistence baseline, thereby providing a clearer and more interpretable measure. The low MASE (0.2285) obtained by RFR suggests strong estimation reliability under conditional inputs and consistency across varying HI levels. The ARIMAX model ranked second (R² = 0.9565), benefitting from the inclusion of exogenous variables that capture external influences on HI. The LSTM model outperformed SARIMAX across all error measures, highlighting its ability to learn nonlinear and sequential dependencies. In contrast, XGBoost showed the weakest performance, with relatively higher errors and the lowest R² (0.8372), suggesting comparatively weaker performance for this dataset and modeling configuration.

Model validation suggests that the conditional estimation results are statistically reliable. Five-fold time-series cross-validation (with chronological folds) showed consistent performance across folds (mean RMSE = 0.89 ± 0.04), indicating good generalizability. Residual diagnostics did not indicate major violations of model assumptions: Durbin–Watson statistics were close to 2, Breusch–Pagan tests yielded non-significant results (p > 0.05), and Q–Q plots displayed near-normal error distributions. These validation results indicate that the conditional estimation framework demonstrates statistical robustness, with all models exhibiting stable generalization behavior and no major assumption violations. Given its superior error metrics, the RFR appears to be the strongest performer, but the overall validation indicates that the model comparisons are robust and unbiased. All evaluated models demonstrated substantially lower scaled error than the naive baseline (MASE < 1), confirming that the proposed framework improves upon simple reference predictions.

#### 3.2.1. Empirical prediction interval performance.

Beyond point-estimation accuracy, predictive uncertainty was evaluated using empirical 95% prediction intervals derived from the distribution of individual tree predictions within the Random Forest ensemble. On the hold-out test set (2022–2023), the 95% empirical prediction intervals achieved a coverage rate of 98.85%, slightly exceeding the nominal level and indicating conservative but reliable uncertainty quantification. The average prediction interval width was 3.94°C, reflecting moderate short-term dispersion consistent with sub-daily climatic variability. These findings suggest that the ensemble-based uncertainty framework provides robust probabilistic characterization of HI estimates while maintaining high point-prediction accuracy.

### 3.3. Scenario-based projections with uncertainty (2024–2027)

**Fig 8:**
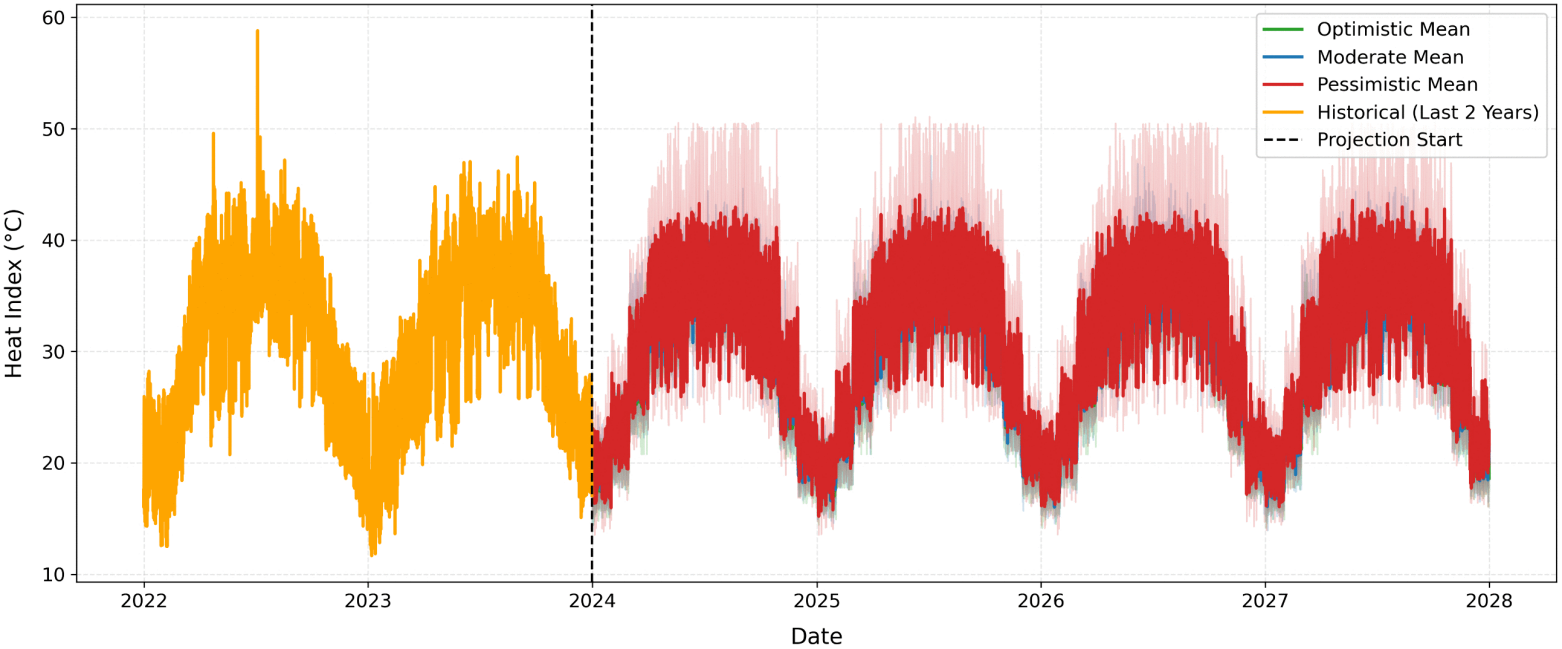
Observed 3-hourly Heat Index (HI) during the test period (2022–2023) and scenario-based projections for 2024–2027 using the Random Forest Regressor. Colored lines represent mean projections under optimistic, moderate, and pessimistic assumptions. Shaded regions denote 95% empirical prediction intervals derived from ensemble tree variability. The vertical dashed line indicates the start of the projection period.

Fig 8 presents the observed 3-hourly HI during the hold-out test period (2022–2023) together with scenario-based projections for 2024–2027 generated using the Random Forest Regressor (RFR). The black line illustrates the observed HI values, which exhibit clear seasonal cycles and high-frequency fluctuations characteristic of Dhaka’s sub-daily climate variability. The smooth transition at the projection boundary reflects the recursive initialization of lagged features using observed 2023 values, ensuring numerical continuity in the scenario simulation. Beyond 2023, three scenario trajectories emerge. The optimistic scenario yields the lowest projected HI levels, the moderate scenario follows an intermediate path, and the pessimistic scenario consistently produces the highest HI values. Across the projection horizon (2024–2027), the average projected HI equals 29.02°C under the optimistic scenario, 29.90°C under the moderate scenario, and 31.33°C under the pessimistic scenario. The approximate 2.31°C difference between optimistic and pessimistic assumptions highlights the sensitivity of apparent temperature dynamics to combined shifts in temperature and humidity.

The shaded bands represent 95% empirical prediction intervals derived from the distribution of individual tree predictions within the ensemble. These uncertainty bands are time-varying and widen notably during peak summer months, reflecting increased predictive dispersion under elevated thermal conditions. In contrast, narrower intervals during winter months indicate greater predictive stability when HI levels are lower. The high empirical coverage observed during validation supports the reliability of these probabilistic projections.

**Fig 9:**
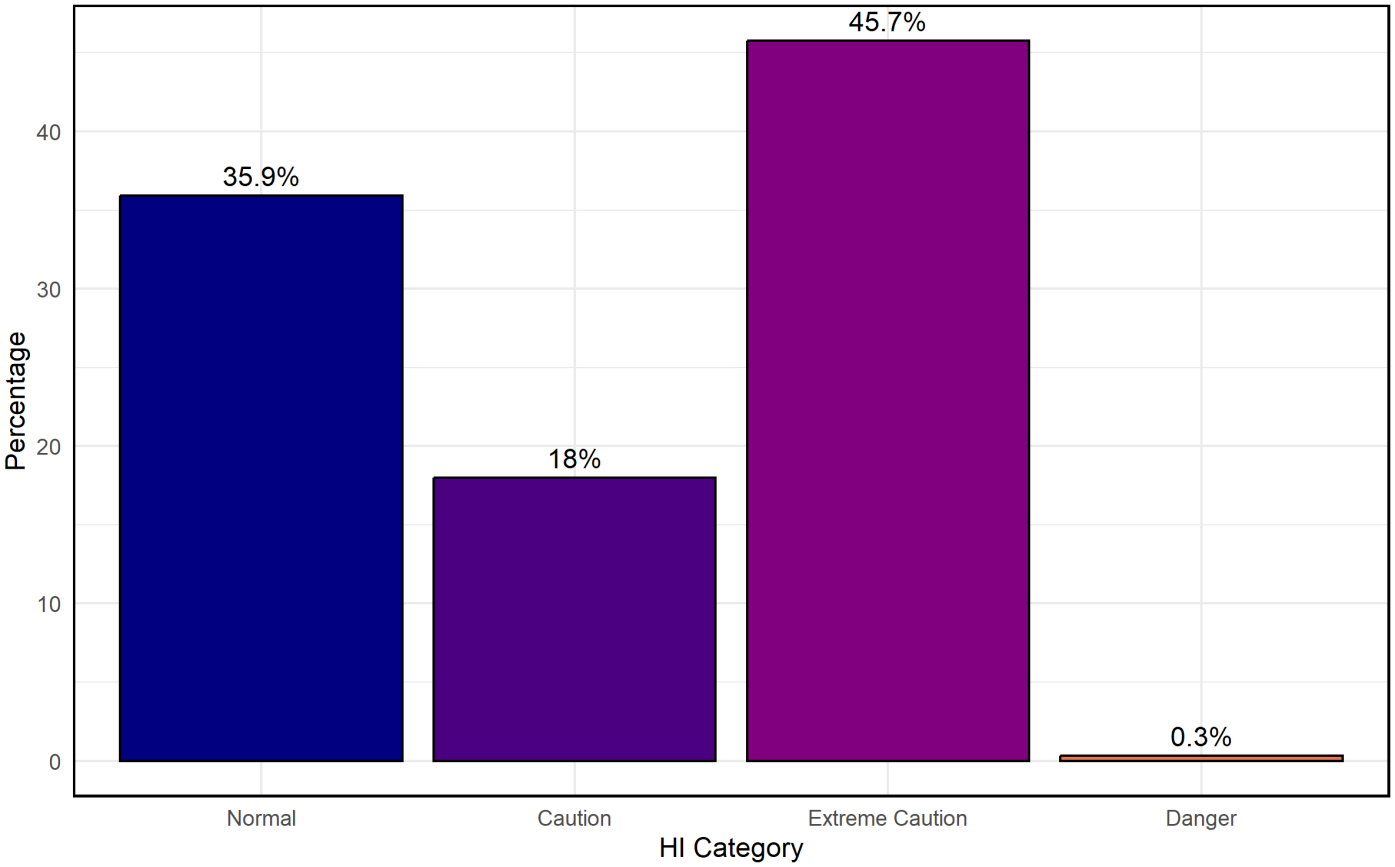
Scenario-based projected distribution of 3-hourly HI risk categories in Dhaka for 2024–2027, highlighting the predominance of sustained “Extreme Caution” conditions relevant for public health preparedness.

To contextualize future heat-related health risks, Fig 9 presents the projected distribution of HI categories from 2024 to 2027 based on 3-hourly scenario projections under the moderate scenario assumption. The largest share of observations (45.7%) falls within the Extreme Caution category, indicating frequent exposure to heat levels associated with elevated risks of heat-related illnesses, particularly during prolonged outdoor activities. The Normal category accounts for 35.9% of observations, while Caution represents 18%, reflecting moderate thermal stress conditions. Only 0.3% of observations reach the Danger category, and none fall into Extreme Danger.

These projections indicate that although the most severe heat extremes appear infrequent within the adopted scenario assumptions, Dhaka may experience sustained periods of moderate to high heat stress. This pattern highlights the importance of targeted public health interventions and urban heat-mitigation strategies, including improved heat-risk communication, early warning systems, and community support mechanisms. Vulnerable groups such as outdoor laborers, children, and the elderly may benefit most from anticipatory actions informed by these projections. Together, the strong performance of the RFR model and the predominance of moderate to high HI categories illustrate the potential relevance of proactive climate-adaptation planning. Urban planning and public health systems could prioritize heat preparedness, particularly during high-risk time windows, to help reduce future impacts.

Finally, heatwaves are characterized by multi-day periods of elevated temperatures. The persistence of Extreme Caution and Caution-level HI values across consecutive 3-hour intervals may be consistent with conditions often associated with prolonged heat stress episodes, although no formal heatwave duration threshold was imposed in this analysis. Such sustained thermal stress may be associated with reduced recovery opportunities between exposure periods. Therefore, identifying and projecting these prolonged moderate-to-high stress patterns can support timely early warnings and effective public health and urban planning interventions.

## 4. Discussion

The accelerating frequency, duration, and intensity of heatwaves under climate change have heightened the need for high-resolution assessment of heat stress dynamics, particularly in densely populated and climate-vulnerable cities such as Dhaka [46,47]. Because the Heat Index (HI) is a deterministic function of air temperature and relative humidity, the framework developed here should be interpreted as a conditional estimation and scenario-projection approach rather than an independent forecasting system. Conventional heatwave definitions based solely on air temperature fail to capture the compounded physiological burden imposed by humidity, which is a dominant driver of thermal discomfort in humid subtropical climates. By adopting the Heat Index (HI) as the primary outcome variable, this study employs a physiologically meaningful metric that more accurately reflects human heat stress and associated health risks, thereby improving upon temperature-only early warning approaches. The primary methodological contribution of this study is the development of a conditional estimation framework that explicitly distinguishes deterministic HI computation from data-driven modeling of its temporal dynamics, enabling scenario-based projections at sub-daily resolution.

A major strength of this study is the use of high-frequency (3-hourly) data spanning a decade. This temporal resolution enables the detection of intra-day variability, nocturnal heat persistence, and short-duration extreme exposures that are typically obscured in daily or monthly datasets. Such fine-scale temporal dynamics are particularly relevant for public health preparedness, as short but repeated periods of elevated heat stress can trigger adverse health outcomes even in the absence of officially declared heatwaves. The observed upward trend and increasing persistence of elevated HI values after 2019 are consistent with documented urban heat island amplification and regional warming, suggesting a growing risk of cumulative thermal stress in Dhaka.

Across all evaluated approaches, Random Forest Regression (RFR) consistently outperformed traditional statistical models (ARIMAX, SARIMAX) as well as other machine-learning methods (XGBoost, LSTM). The superior performance of RFR is likely related to its capacity to capture nonlinear interactions and threshold effects between temperature, humidity, diurnal cycles, and seasonal variability without imposing parametric assumptions [48]. HI dynamics are inherently nonlinear, particularly near physiological risk thresholds. In these conditions, small increases in humidity can disproportionately amplify perceived heat stress. Tree-based ensemble methods naturally partition the predictor space around such nonlinearities, enabling more accurate representation of heat stress escalation than linear time-series models that rely on stationarity assumptions. The comparatively weaker performance of ARIMAX and SARIMAX models highlights the limitations of linear frameworks in representing high-frequency HI variability. While these models effectively capture seasonal structure and autocorrelation, they are less capable of modeling abrupt humidity-driven amplification effects that dominate tropical urban heat stress. Similar limitations have been reported in prior heat index studies using daily-scale statistical models, where seasonal trends are well represented but sub-daily extremes are smoothed out. The empirical prediction intervals demonstrated robust calibration performance. The slightly conservative coverage (98.85%) indicates that the ensemble-based approach adequately captures predictive dispersion without underestimating uncertainty. Reliable uncertainty quantification is particularly important in public health contexts, where underestimation of risk may have serious consequences.

Comparisons with closely related studies further contextualize these findings. Yodpibul et al. (2021) demonstrated improved conditional HI estimation performance in Thailand using daily-scale autocorrelation-based models; however, their framework did not explicitly address intra-day variability [24]. Our results extend this line of work by showing that high-frequency (3-hourly) modeling reveals additional risk information associated with the persistence of elevated HI values within a single day—an aspect that is particularly relevant for occupational and public health interventions. Deep learning approaches such as LSTM have shown strong performance in several climate and urban heat studies, particularly when applied to smoother, lower-frequency time series [49,50]. Lateef et al. (2021) and Uppal et al. (2021), for example, reported improved short-term conditional HI estimation using neural networks architectures under relatively stable meteorological conditions [25,26]. In contrast, our findings indicate that LSTM underperforms in highly volatile, high-resolution HI data. This discrepancy is likely mechanistic: LSTM models require stable temporal patterns and large effective sample sizes to reliably learn long-term dependencies, whereas rapid temperature–humidity interactions in tropical urban climates introduce noise that limits their generalization capacity. These results reinforce the growing consensus that deep learning superiority in environmental forecasting is strongly context-dependent rather than universal.

Recent work by Qureshi et al. (2025) proposed a seasonal-adjusted hybrid machine-learning framework for heatwave warning in Bangladesh using daily data [47]. While their approach effectively addressed seasonal non-stationarity, the present study advances this framework by demonstrating that ensemble learning at sub-daily resolution conditionally captures additional dimensions of heat risk. Specifically, we show that prolonged sequences of “Extreme Caution” HI conditions, even without frequent “Danger”-level extremes, may be associated with substantial cumulative physiological strain. This persistence-based risk mechanism is not readily detectable in daily-scale analyses but is critical for understanding cumulative exposure in humid megacities.

The comparatively weaker performance of XGBoost in this study further highlights the challenges of modeling high-frequency environmental time series. Although gradient boosting methods have demonstrated success in various climate applications, their performance may degrade under conditions of strong temporal autocorrelation and rapid intra-day variability. This finding aligns with recent methodological studies suggesting that boosting algorithms can struggle in noise-dominated, high-resolution settings unless explicitly tailored for temporal dependence [51].

Under the moderate scenario assumption, projections for 2024–2027 indicate that a substantial proportion of future 3-hourly observations may fall within the “Extreme Caution” HI category. Although the most severe categories (“Danger” and “Extreme Danger”) remain relatively rare, the persistence of moderate-to-high HI levels across consecutive time intervals is of particular concern. Sustained thermal stress may limit opportunities for physiological recovery between exposure periods and can significantly elevate health risks among vulnerable populations, even in the absence of extreme peak values. These findings emphasize the importance of incorporating persistence and duration, rather than isolated extremes alone, into heat-health warning systems.

The extended projections should be interpreted as scenario-based projections rather than deterministic predictions. Since HI values are deterministically derived from temperature and humidity, the projected outcomes depend strongly on the assumed meteorological inputs rather than representing fully independent climate forecasts. Accordingly, the added value of the proposed framework lies not in redefining the HI formula but in capturing its nonlinear temporal persistence and translating conditional meteorological scenarios into actionable sub-daily health-relevant projections. This distinction is critical to avoid misinterpretation of machine-learning outputs as independent physical forecasts. Future temperature and humidity inputs were derived from historical monthly averages, preserving diurnal and seasonal structure while not explicitly accounting for long-term climate change trajectories or extreme anomaly events. Consequently, uncertainty is expected to increase with projection horizon due to recursive error propagation and reliance on climatological predictor assumptions. Nonetheless, such scenario-based projections remain valuable for anticipatory planning in data-constrained settings, where near-term operational guidance is often more actionable than long-range climate projections. Therefore, the proposed framework should be interpreted as a scenario-exploration tool rather than a deterministic forecasting system, with its primary value lying in translating meteorological assumptions into health-relevant heat-stress insights.

From a policy and public health perspective, the high temporal resolution of the proposed conditional estimation framework offers distinct operational advantages. Hour-specific HI estimates and scenario-based projections can inform time-targeted interventions, including adjustments to outdoor labor schedules, issuance of heat advisories during peak risk periods, and optimized deployment of cooling and emergency response resources. Integrating such forecasts into urban heat action plans could substantially enhance the precision and effectiveness of heat-risk mitigation strategies in Dhaka. Despite its strengths, this study has limitations that warrant consideration. The exclusion of additional environmental variables such as wind speed, solar radiation, land-use characteristics, and urban morphology may constrain the model’s ability to capture localized heat stress variability. Furthermore, reliance on historical averages for future predictor generation limits sensitivity to accelerating climate change. Future research should incorporate downscaled climate projections, remote-sensing–derived urban indicators, and probabilistic climate-informed predictor ensembles to enhance both realism and uncertainty characterization.

## 5. Conclusions

This study demonstrates the potential value of a high-resolution machine-learning framework for conditional estimation and scenario-based projection of the Heat Index (HI) to support heat stress management in Dhaka. Under the moderate scenario assumption, projected HI values predominantly fall within the “Extreme Caution” category, indicating sustained moderate heat-stress conditions rather than frequent extreme peaks. These findings highlight the potential value of anticipatory planning to protect vulnerable populations, particularly older adults, children, and outdoor workers.

The results show that ensemble-based models, particularly Random Forest Regression, appear well suited for capturing the nonlinear and high-frequency dynamics of heat stress in humid tropical cities. Sub-daily HI scenario projections provide actionable temporal insights for targeted interventions, including heat early warning systems, heat action plans, community cooling centers, and public awareness initiatives. These applications are consistent with global anticipatory action frameworks, including WHO and UNDRR guidance [52,53]. Because HI is deterministically derived from temperature and relative humidity, the projected outcomes depend on assumed meteorological inputs and should complement rather than replace dedicated weather or climate forecasting systems. Despite these strengths, projection uncertainty is expected to increase with lead time due to reliance on historical climatological patterns, and model performance may be sensitive to data resolution and parameterization. Future work integrating additional environmental variables and climate-informed projections would further strengthen forecast robustness. Overall, this study provides a reproducible and policy-relevant framework for conditional heat-stress assessment and anticipatory planning in rapidly urbanizing and climate-vulnerable settings.

## Supporting information

S1 File Dataset of 3-hourly meteorological observations (2014–2023). (XLSX)
