# Supplementary material for: Machine-learning framework for conditional estimation and scenario-based projection of the heat index for public health interventions
Source: PLoS One. 2026 Apr 28;21(4):e0348202. doi: 10.1371/journal.pone.0348202 (PMC13123980; doi:10.1371/journal.pone.0348202)
Supplement: Attachment — Submitted filename: Response to Reviewers.pdf [file pone.0348202.s002.pdf]

24th October, 2025

Forecasting Heat Index for Proactive Public Health Interventions: A Machine Learning Framework with Exogenous Predictors (Manuscript ID: PONE-D-25-44349)

Dear Editor,

We sincerely thank you and the reviewers for your time and thoughtful comments on our manuscript. We appreciate the opportunity to revise our work and have carefully addressed each concern to enhance the scientific quality, clarity, and structure of the manuscript. Below, we provide a detailed, point-by-point response to all the editorial and reviewer comments.

Best regards,

Authors

Editorial Requirements

1. When submitting your revision, we need you to address these additional requirements. Please ensure that your manuscript meets PLOS ONE's style requirements, including those for file naming. The PLOS ONE style templates can be found at https://journals.plos.org/plosone/s/file?id=wjVg/PLOSOne_formatting_sample_main_body.pdf and https://journals.plos.org/plosone/s/file?id=ba62/PLOSOne_formatting_sample_title_authors_affiliations.pdf

Author's response:
We have carefully reviewed the PLOS ONE style templates and updated the manuscript to comply with all formatting requirements, including file naming conventions. All sections, headings, references, and figures now adhere to the journal's style guidelines.

2. Please note that PLOS One has specific guidelines on code sharing for submissions in which author-generated code underpins the findings in the manuscript. In these cases, we expect all author-generated code to be made available without restrictions upon publication of the work. Please review our guidelines at https://journals.plos.org/plosone/s/materials-and-software-sharing#loc-sharing-code and ensure that your code is shared in a way that follows best practice and facilitates reproducibility and reuse.

Author's response:
We will share all author-generated code without restrictions in a public GitHub repository upon manuscript acceptance. The repository will be cited in the manuscript, include a detailed README file, and be archived on Zenodo to provide a permanent DOI. We confirm adherence to PLOS ONE's code sharing guidelines.

3. Please include captions for your Supporting Information files at the end of your manuscript, and update any in-text citations to match accordingly. Please see our Supporting Information guidelines for more information: http://journals.plos.org/plosone/s/supporting-information.

Author's response:

Captions for all Supporting Information files have been added at the end of the manuscript. All in-text references to Supporting Information have been updated to match these captions, in accordance with the journal's guidelines.

4. If the reviewer comments include a recommendation to cite specific previously published works, please review and evaluate these publications to determine whether they are relevant and should be cited. There is no requirement to cite these works unless the editor has indicated otherwise.

Author's response:
We have reviewed the publications recommended by the reviewers and evaluated their relevance to our study. Relevant works have been cited appropriately in the revised manuscript; citations unrelated to the study objectives have been omitted, consistent with the editor's guidance.

Reviewer #1 Comments

This manuscript examines a critical and globally pertinent issue: the enhancement of urban waste management through the integration of AI and IoT systems. The theme is pertinent and strongly aligns with the increasing advocacy for intelligent, sustainable urban environments. I value how the paper frames waste management as not merely a logistical challenge but as a fundamental sustainability issue that intersects with urban health, environmental protection, and effective governance. The aspiration to incorporate AI and IoT into this discourse is laudable, and the prospective influence of these technologies is indisputable; instances like Barcelona's intelligent waste bins and Seoul's RFID-enabled food waste monitoring system demonstrate how data-centric systems can diminish operational expenses and enhance recycling rates.

Nonetheless, I observed that the manuscript resembles a conceptual overview rather than a methodically organized research paper. The originality of the work remains ambiguous: does it present a novel framework, enhance existing AI-IoT models, or primarily constitute a literature synthesis? For instance, the paper references the predictive monitoring of waste levels via IoT sensors, a concept well-documented in previous research (e.g., experiments utilizing ultrasonic sensor-based smart bins in Singapore). Your study must clearly articulate its unique perspective, whether by introducing an enhanced algorithm, examining a novel city-scale case study, or providing a comparative analysis of models.

The methodology section, in particular, requires significantly enhanced clarity. Currently, it is unclear whether the work relies on simulations, case studies, or an analysis of existing implementations. For example, if you assert efficiency improvements, are these derived from machine learning simulations (such as a neural network forecasting waste generation patterns) or based on pilot project data? Elucidating the selection of algorithms, be it support vector machines, random forests, or reinforcement learning, and their interaction with IoT systems is essential for readers to evaluate the technical integrity of the work. The absence of transparency restricts the study's reproducibility.

Likewise, the findings are presently articulated in general, nearly aspirational language. Assertions like "AI-driven IoT can enhance sustainability outcomes" are persuasive in theory

but necessitate empirical validation. Consider incorporating case-based data: for instance, research in Sweden indicated that AI-assisted routing for waste trucks decreased fuel consumption by nearly 20%, while pilot projects in India revealed that real-time bin monitoring enhanced collection efficiency in densely populated informal settlements. Incorporating comparative evidence would enhance your argument and underscore the practical viability of your proposals.

The implications for policy and practice warrant greater emphasis. Municipalities, particularly in low- and middle-income nations, encounter obstacles in implementing AI-IoT systems, including substantial initial costs, disjointed governance, and restricted technical capabilities. Elaborating on the phased implementation of your model, beginning with high-density business districts and subsequently expanding to encompass entire cities, would enhance its practical relevance. Furthermore, sustainability encompasses not only efficiency but also equity. In what ways could marginalized neighborhoods gain advantages from or be marginalized by these technologies? Incorporating this dimension would enhance the paper's social significance.

From a presentation standpoint, the manuscript is comprehensible but requires refinement. Numerous sections employ lengthy, redundant sentences, where more succinct phrasing would enhance coherence. Incorporating a schematic diagram of the proposed AI-IoT architecture would enhance readers' comprehension of the interactions among sensors, data analytics, and decision-making within the system. Likewise, tables that encapsulate current global implementations of smart waste initiatives could contextualize your research within the wider domain.

Author’s response:
We sincerely thank Reviewer 1 for the time and effort devoted to reviewing our submission. However, upon careful reading, we believe that these comments may not relate to our manuscript entitled “Forecasting Heat Index for Proactive Public Health Interventions: A Machine Learning Framework with Exogenous Predictors.”

Reviewer 1’s feedback discusses AI- and IoT-based smart waste management systems, which appear to belong to another manuscript. Since our study focuses specifically on heat index forecasting using meteorological data (temperature, humidity, etc.), we could not identify a way to incorporate these comments without deviating from our research scope.

Accordingly, we respectfully note this possible mismatch for the editor’s attention. We remain committed to revising our manuscript in line with the relevant methodological and presentation concerns raised by Reviewer 2, and we have fully addressed those points in the revised version.

Reviewer #2 Comments

The manuscript addresses an important public health and climate adaptation challenge: forecasting the Heat Index (HI) for Dhaka using machine learning and statistical models. The topic is timely and relevant considering the increasing frequency of heatwaves in South Asia. The paper is well-written and organized, but various methodological problems need to be fixed and clarified before it can be published.

Abstract:

1. The abstract reports exact evaluation metrics (R^2, MAE, RMSE, etc.), which is too detailed for this section. Please highlight relative performance instead (e.g., "Random Forest outperformed other models").

Author's response:
We thank the reviewer for the suggestion. The abstract has been revised to focus on relative model performance rather than reporting exact numerical metrics. The Random Forest Regressor (RFR) is now described as achieving the highest predictive accuracy among all models, outperforming both statistical and deep learning approaches, while preserving clarity for the abstract section.

2. How exactly did you forecast to 2027? What data did you use for future temperature and humidity? This isn't clear and needs explanation.

Author's response:
We thank the reviewer for this important observation. To forecast the Heat Index for 2024–2027, we used our trained Random Forest model, which requires temperature and humidity as input features. As actual meteorological data beyond 2023 were unavailable, we estimated future temperature and humidity using historical monthly averages from 2014–2023. These averages were repeated across the forecast horizon at 3-hour intervals to maintain seasonal and diurnal patterns. Small random variations were also added to temperature, humidity, and predicted Heat Index values to mimic real-world variability. This approach allowed the model to generate indicative forecasts reflecting typical seasonal dynamics rather than exact future values. The Abstract has been updated to briefly mention this procedure, and full methodological details can be found in **Section 2.5.6 "Preparation of Future Predictor Variables for Forecasting"** of the revised manuscript.

3. The policy and public health implications are appropriate to include, but the language should be softened to avoid overstatement. Consider phrases like this "This framework could potentially support early warnings …".

Author's response:
We thank the reviewer for the suggestion. The conclusion in the abstract has been revised to use more measured language. Phrases such as "could potentially support early warning systems" and "guide adaptive urban heat management strategies" have been adopted to convey practical implications without overstating certainty.

Introduction:

1. You repeat some points (e.g., reliance on temperature-only warnings; global increase in heatwave days)

Author's response:
We thank the reviewer for pointing this out. The introduction has been revised to remove redundancy, consolidating mentions of temperature-only warnings and global heatwave trends into single, clear passages. The revised text now presents a concise, coherent narrative from

global to local context, highlighting the research gap and objectives without repeating points.

Methods:

1. Please clarify if any gaps existed in the dataset and how they were handled; were they excluded, interpolated, etc.?

Author's response:
We have explicitly noted that data completeness was verified during preprocessing and no missing values were found after validation. A clarifying sentence was added in **Section 2.2**.

2. Using a random 80/20 split for time series data creates data leakage - the model sees future patterns when training. You need to split by time periods instead.

Author's response:
We thank the reviewer for the comment. The statement in the manuscript describing an 80/20 split was a textual mistake. In the analysis, the dataset was correctly split chronologically by time periods, with data from 2014–2021 used for training and 2022–2023 for testing. The manuscript has been corrected to reflect this in **Section 2.4**.

3. The future forecasting (2024–2027) methodology is fundamentally unclear. RF and XGBoost require exogenous predictors (temperature, humidity) that don't exist for future years. How were these obtained? If you only used seasonal patterns, say so and explain why that limits how useful the forecasts are.

Author's response:
We thank the reviewer for this important observation. To clarify, we have added a new subsection in the Materials and Methods titled **"2.5.6 Preparation of Future Predictor Variables for Forecasting"**, which details how future exogenous variables were generated for all forecasting models. Specifically, temperature and humidity for 2024–2027 were approximated using historical monthly averages from 2014–2023, which were repeated at 3-hourly intervals to preserve seasonal and diurnal patterns. For machine learning models such as Random Forest, XGBoost, and LSTM, small random variations were introduced to mimic realistic variability, while statistical models (ARIMAX and SARIMAX) used the deterministic monthly averages directly.

We note that this approach assumes that near-future climatic conditions will follow historical seasonal cycles. As a result, the forecasts should be interpreted as **scenario-based projections** rather than precise predictions, and they do not capture potential long-term climate changes or anomalies. This limitation is now explicitly stated in the subsection to ensure transparency regarding the scope and interpretability of the future forecasts.

Results:

1. The Random Forest results (R^2 = 0.9860, MAPE = 0.02%) are unusually high for environmental forecasting. This suggests overfitting or that your validation method has problems.

Author's response:
We thank the reviewer for the comment. The previously reported metrics were a reporting mistake. The correct metrics for the Random Forest model on the chronological test set are:

- $R^2 = 0.9872$
- MAE = 0.5485
- RMSE = 0.8473
- MAPE = 1.71%
- MASE = 0.2285

The high R^2 reflects the inclusion of lag features (*HeatIndex_lag_1*, *lag_3*, *lag_6*) and time-based features (*Hour*, *Day*, *Month*, *Season*), which capture strong short-term autocorrelation and seasonal patterns in the Heat Index. The model was evaluated on a ***chronological test set***, so no future information was used in training. We have clarified this in the revised manuscript to prevent confusion regarding overfitting.

2. MAPE values of 0.02-0.08% seem artificially low. With high heat index values, percentage errors naturally look small even when absolute errors aren't. Consider more robust error metrics.

Author's response:
We thank the reviewer for highlighting this. The previously reported MAPE values were due to a reporting mistake; the correct MAPE is 1.71%. To provide a more robust evaluation of model performance, we have now included MASE (Mean Absolute Scaled Error), which was not part of the original manuscript. The metrics table has been fully updated to report MAE, RMSE, MAPE, R^2, and MASE. MASE compares the model's prediction errors to a naive forecast, making it less sensitive to high Heat Index values and providing a clearer assessment of predictive accuracy. This addition ensures that absolute and relative errors are transparently reported.

Discussion:
1. Keep the Discussion focused on interpreting results and move broader implications, limitations, and future research into a separate section (e.g. "Conclusion")

Author's response:
We sincerely thank the reviewer for this valuable suggestion. In the revised manuscript, the Discussion section has been refined to focus exclusively on interpreting model results, comparing performance metrics, and linking findings with relevant literature. Broader implications, study limitations, and directions for future research have been moved to a newly structured Conclusion section **(Section 5)**. This restructuring improves the clarity, focus, and logical organization of the manuscript.
