# Supplementary material for: Machine-learning framework for conditional estimation and scenario-based projection of the heat index for public health interventions
Source: PLoS One. 2026 Apr 28;21(4):e0348202. doi: 10.1371/journal.pone.0348202 (PMC13123980; doi:10.1371/journal.pone.0348202)
Supplement: Attachment — Submitted filename: Response_to_Reviewers_R2.pdf [file pone.0348202.s003.pdf]

4th December, 2025

Forecasting Heat Index for Proactive Public Health Interventions: A Machine Learning Framework with Exogenous Predictors (Manuscript ID: PONE-D-25-44349R1)

Dear Editor,

We sincerely thank you and the reviewers for your time and thoughtful comments on our manuscript. We appreciate the opportunity to revise our work and have carefully addressed each concern to enhance the scientific quality, clarity, and structure of the manuscript. Below, we provide a detailed, point-by-point response to all the editorial and reviewer comments.

Best regards,

Authors

Journal Requirements

If the reviewer comments include a recommendation to cite specific previously published works, please review and evaluate these publications to determine whether they are relevant and should be cited. There is no requirement to cite these works unless the editor has indicated otherwise.

Author's Response:
While the reviewer did not suggest specific publications, we have added several relevant references in the Discussion section of the revised manuscript to provide additional context and support.

Reviewer #1 Comments

The study offers a valuable analysis of environmental and climatic interactions using remote sensing data and statistical methods, but it needs considerable enhancement before acceptance. Although the topic is timely and the datasets are suitable, the methodological details, such as data preprocessing, model validation, and statistical robustness, are insufficiently explained. The discussion often repeats results without deeper interpretation or connection to existing literature, and causal inferences are drawn without sufficient evidence. The manuscript also requires thorough language editing to improve clarity and readability. Overall, the research shows promise but must undergo significant revision to strengthen its scientific rigour, transparency, and presentation quality.

Author's Response:
We break the whole comments into points to provide a clear answer to each of them. The point-by-point responses are given below.

1. "The methodological details, such as data preprocessing, model validation, and statistical robustness, are insufficiently explained."

Response:
Thank you for pointing this out. We have substantially expanded the Methods section to enhance transparency and reproducibility. In particular:

- A comprehensive, step-by-step preprocessing workflow, covering format conversion, dataset merging, timestamp validation, missing-value assessment, and outlier verification has been added in **Section 2.2.**
- Detailed explanations of model validation procedures and statistical robustness checks have been incorporated into a newly developed **Section 2.6.**

These revisions greatly improve the methodological clarity and completeness of the manuscript.

2. "The discussion often repeats results without deeper interpretation or connection to existing literature, and causal inferences are drawn without sufficient evidence."

Response:
We appreciate this insightful observation. The Discussion section has been thoroughly rewritten to:

- Provide deeper interpretation rather than repeating numerical findings
- Connect the results with relevant and recent literature on heatwave forecasting, machine-learning-based climate models, and urban heat stress
- Clarify why the RFR model performed best
- Discuss implications for public health risk, urban planning, and climate-adaptation strategies in Dhaka
- Remove speculative language and avoid unsupported causal interpretations

These changes enhance the scholarly depth, relevance, and analytical quality of the Discussion.

3. "The manuscript requires thorough language editing to improve clarity and readability."

Response:
The entire manuscript has been carefully edited for coherence, academic tone, grammar, and clarity. Long sentences were shortened, redundancies removed, and transitions improved. The revised version adheres to scientific writing standards and improves overall readability. Changes applied throughout the manuscript.
