# Supplementary material for: Machine-learning framework for conditional estimation and scenario-based projection of the heat index for public health interventions
Source: PLoS One. 2026 Apr 28;21(4):e0348202. doi: 10.1371/journal.pone.0348202 (PMC13123980; doi:10.1371/journal.pone.0348202)
Supplement: Attachment — Submitted filename: Response to Reviewers_03.pdf [file pone.0348202.s004.pdf]

9th January, 2026

Forecasting Heat Index for Proactive Public Health Interventions: A Machine Learning Framework with Exogenous Predictors (Manuscript ID: PONE-D-25-44349R2)

Dear Editor,

We sincerely thank you and the reviewers for their time, careful evaluation, and constructive comments on our manuscript. We appreciate the opportunity to revise our work and have carefully addressed all concerns to improve the methodological rigor, interpretative depth, clarity, and overall presentation of the study. Below, we provide a detailed point-by-point response to the reviewer's comments, indicating how each issue has been addressed in the revised manuscript.

Kind regards,

Authors

Journal Requirements

If the reviewer comments include a recommendation to cite specific previously published works, please review and evaluate these publications to determine whether they are relevant and should be cited. There is no requirement to cite these works unless the editor has indicated otherwise.

Author's Response:
No specific publications were recommended for citation by the reviewer. Therefore, no additional references were added.

Reviewer #1 Comments

The revised manuscript demonstrates notable improvement in comparison to its previous iteration, especially concerning the structure and elucidation of research objectives. The subject matter remains pertinent, and the datasets employed are suitable for the scope of the investigation. Nonetheless, several fundamental issues warrant further attention prior to the work's publication readiness.

Although the methodological framework is largely appropriate, key analytical procedures require more comprehensive justification. While statistical procedures and parameter selections are articulated with greater clarity than previously, validation processes, uncertainty analyses, and robustness assessments remain somewhat limited. Consequently, some conclusions are presented with a strength that may not be fully supported by the data. The discussion section has been expanded; however, it still predominantly relies on descriptive interpretation rather than providing deeper mechanistic explanations or comparisons with closely related studies.

The manuscript is comprehensible; however, the quality of English language usage necessitates further refinement. Recurring grammatical errors, lengthy sentences, and redundant phrases detract from clarity and readability. Figures and tables are informative but would benefit from more precise captions and a clearer connection to the analytical narrative.

In summary, the study possesses considerable potential, but additional revisions are imperative to enhance methodological rigor, improve interpretative clarity, and refine linguistic precision.

Author's Response:

We break the whole comments into points to provide a clear answer to each of them. The point-by-point responses are given below.

1. "Although the methodological framework is largely appropriate, key analytical procedures require more comprehensive justification."

Response:

We thank the reviewer for this insightful comment. In the revised manuscript, we have substantially strengthened the justification of the analytical framework. Specifically, **Section 2.5** now clearly explains the rationale for selecting ARIMAX and SARIMAX as statistical benchmarks, Random Forest and XGBoost as nonlinear ensemble learners, and LSTM as a deep learning approach for sequential dependency modeling. The motivation for using high-frequency (3-hourly) data and the Heat Index as a physiologically meaningful outcome variable has also been expanded. These revisions clarify the methodological choices and their relevance to urban heat-stress forecasting.

2. "Validation processes, uncertainty analyses, and robustness assessments remain somewhat limited."

Response:

We thank the reviewer for this important observation. While a validation section was included in the previous version of the manuscript, **Section 2.6 (Model Validation and Robustness Assessment)** has now been substantially strengthened and refined in direct response to this comment.

In the revised manuscript, we clarify and expand the validation framework by explicitly describing the use of five-fold time-series cross-validation with strict preservation of chronological order to prevent information leakage and ensure realistic forecasting evaluation. The procedures for hyperparameter optimization are now described more clearly, and the role of early stopping in improving generalization for deep learning models is explicitly stated.

For the statistical models (ARIMAX and SARIMAX), the description of residual diagnostics has been refined to clearly document the assessment of autocorrelation, serial dependence, heteroscedasticity, and distributional conformity using autocorrelation and partial autocorrelation functions, Durbin–Watson statistics, Breusch–Pagan tests, and Q–Q plots. The revised text explicitly notes that these diagnostics did not indicate substantial violations of modeling assumptions.

In addition, we now explicitly address forecast robustness and uncertainty, noting that model performance was stable across cross-validation folds and clarifying that future projections are scenario-based estimates derived from climatological averages. This revision strengthens transparency regarding uncertainty while avoiding overstatement of predictive certainty.

We believe these revisions substantially improve the rigor, clarity, and completeness of the validation and robustness assessment and directly address the reviewer's concern.

3. "Some conclusions are presented with a strength that may not be fully supported by the data."

Response:
We thank the reviewer for highlighting this concern. In response, we have carefully revised the language throughout the Results, Discussion, and Conclusions sections to ensure appropriate scientific caution. Forecasts for the 2024–2027 period are now explicitly described as scenario-based projections, derived from climatological averages rather than climate-change-driven simulations. Statements implying deterministic prediction have been removed or softened, and uncertainty with increasing lead time is clearly acknowledged. These revisions ensure that the strength of the conclusions is fully aligned with the supporting evidence.

4. "The discussion section remains largely descriptive and would benefit from deeper mechanistic explanations and comparison with related studies."

Response:
We appreciate this valuable suggestion. The Discussion section has been substantially expanded to include mechanistic interpretations of model performance. In particular, we explain why Random Forest Regression outperformed other approaches by highlighting its ability to capture nonlinear temperature–humidity interactions and threshold effects inherent in Heat Index dynamics. The comparatively weaker performance of LSTM and XGBoost is discussed in the context of high-frequency, noise-dominated climatic time series.

We have also strengthened comparisons with closely related studies, including prior work on Heat Index forecasting and heatwave modeling in South Asia and comparable climates. These additions move the discussion beyond descriptive interpretation and provide a deeper process-based understanding of the results.

5. "The quality of English language usage requires further refinement, and figures and tables would benefit from more precise captions and clearer linkage to the analytical narrative."

Response:
We thank the reviewer for this comment. The manuscript has undergone careful language polishing to improve clarity, conciseness, and readability. Long sentences have been shortened, redundant phrasing removed, and grammatical consistency improved throughout the text.

Figure and table captions have been revised to be more precise and informative, explicitly linking visual outputs to their analytical and public-health relevance. The revised captions now clearly describe the content, interpretation, and significance of each figure and table within the broader narrative.
