# Supplementary material for: Machine-learning framework for conditional estimation and scenario-based projection of the heat index for public health interventions
Source: PLoS One. 2026 Apr 28;21(4):e0348202. doi: 10.1371/journal.pone.0348202 (PMC13123980; doi:10.1371/journal.pone.0348202)
Supplement: Attachment — Submitted filename: Response_to_Reviewer_04.pdf [file pone.0348202.s005.pdf]

March 04, 2026

Machine-Learning Framework for Conditional Estimation and Scenario-Based Projection of the Heat Index for Public Health Interventions (Manuscript ID: PONE-D-25-44349R3)

Dear Editor,

We sincerely thank you and the reviewer for the careful evaluation of our manuscript and for the constructive comments that have helped us substantially improve the conceptual clarity, methodological rigor, and transparency of our work. We have carefully revised the manuscript to address all concerns. Below, we provide a detailed point-by-point response outlining how each issue has been resolved in the revised version.

Kind regards,

Authors

Journal Requirements

If the reviewer comments include a recommendation to cite specific previously published works, please review and evaluate these publications to determine whether they are relevant and should be cited. There is no requirement to cite these works unless the editor has indicated otherwise.

Author's Response:

No specific publications were recommended for citation by the reviewer. Therefore, no additional references were added.

Reviewer #1 Comments

The manuscript addresses a significant and timely public health concern by examining the dynamics of the Heat Index through the utilization of high-frequency meteorological data and multiple modeling approaches. The overall motivation is well-articulated, and the comparative modeling efforts constitute a notable positive aspect. However, despite enhancements over earlier versions, several critical issues persist that undermine the manuscript's technical robustness.

A primary concern pertains to the conceptual framing of the study. The Heat Index is a deterministic function of temperature and relative humidity; nevertheless, the manuscript continues to portray the task as "forecasting" the Heat Index without explicitly clarifying the operational assumptions involved. The contribution would be considerably strengthened if the authors explicitly contextualized the work as conditional Heat Index estimation based on assumed future meteorological inputs or, alternatively, concentrated on forecasting temperature and humidity prior to deriving the Heat Index.

Furthermore, the statistical rigor requires reinforcement. Key details regarding forecasting horizons, measures to prevent data leakage when utilizing lagged variables, comparisons with

baseline models, and methods for quantifying uncertainty are either omitted or insufficiently addressed. The long-term analysis (2024–2027), which relies on historical averages supplemented with random perturbations, limits the model's capacity to accurately represent extreme events and diminishes the robustness of the quantitative conclusions derived from these scenarios.

In addition, data availability does not fully comply with publication guidelines, given that it is unclear whether all processed datasets and analysis codes are publicly accessible. In summary, while the study demonstrates considerable potential, it necessitates further revision to improve conceptual clarity, methodological rigor, data transparency, and presentation quality.

Author's Response:

We break the whole comments into points to provide a clear answer to each of them. The point-by-point responses are given below.

1. Conceptual Framing of Heat Index "Forecasting"

Comment:

The Heat Index (HI) is a deterministic function of temperature and relative humidity; therefore, portraying the task as "forecasting" HI requires clearer operational assumptions.

Response:

We fully agree and have substantially clarified the conceptual framing throughout the manuscript. The following revisions were implemented:

- The manuscript title has been revised from **"Forecasting Heat Index for Proactive Public Health Interventions: A Machine Learning Framework with Exogenous Predictors"** to **"Machine-Learning Framework for Conditional Estimation and Scenario-Based Projection of the Heat Index for Public Health Interventions"** to reflect the clarified conceptual framing.
- We explicitly state throughout the manuscript that HI is deterministically derived from air temperature and relative humidity.
- The modeling task is now consistently described as conditional estimation under assumed meteorological inputs rather than independent climate forecasting.
- The Discussion clarifies that the added value of the framework lies in capturing nonlinear temporal persistence and translating conditional meteorological scenarios into actionable sub-daily projections.

These changes ensure conceptual precision and prevent misinterpretation regarding deterministic properties of HI.

2. Forecasting Horizon and Prevention of Data Leakage
Comment:
Clarification was requested regarding forecasting horizons and safeguards against data leakage when using lagged variables.

Response:
We strengthened Sections 2.4, 2.5, and 2.6 to clarify:

- Training period: 2014–2021
- Test period: 2022–2023
- Recursive 3-hour-ahead conditional estimation framework
- Strict chronological partitioning
- Expanding-window five-fold time-series cross-validation

We explicitly state that:

- All lagged HI predictors were generated strictly from past observations within each temporal partition.
- Feature scaling was performed using training data only.
- No information from the test period was used during feature construction.

These revisions eliminate potential data leakage concerns.

3. Baseline Model Comparison
Comment:
Inclusion of a baseline model for comparison was recommended.

Response:
We incorporated a naïve persistence benchmark ($HI_t = HI_{t-1}$) and evaluated performance using MAE, RMSE, R^2, and MASE. The inclusion of MASE provides a scale-independent comparison relative to the naive baseline. All evaluated models achieved MASE < 1, confirming improved performance over the persistence benchmark. This strengthens the comparative rigor of the study.

4. Uncertainty Quantification
Comment:
Greater clarity was requested regarding uncertainty estimation.

Response:
We expanded uncertainty quantification by:

- Implementing empirical 95% prediction intervals derived from ensemble tree dispersion.
- Adding a new subsection: "**2.5.7 Empirical Tree-Based Prediction Intervals**"
- Reporting empirical coverage probability (98.85%) and average interval width (3.94°C).

- Adding an **“Empirical Prediction Interval Performance”** subsection in Results (**Section 3.2**).
- Revising **Section 2.6** (**Model Validation and Robustness Assessment**).

We emphasize that prediction intervals quantify conditional model dispersion under fixed meteorological inputs and do not incorporate structural climate-model uncertainty. These revisions substantially improve statistical transparency.

5. Long-Term Projections (2024–2027)
Comment:
The long-term scenario construction based on historical averages may limit representation of extreme events.

Response:
We revised the projection framework to clearly define these outputs as scenario-based climatological projections, not climate-change forecasts.
Revisions include:

- Explicit clarification that future temperature and humidity inputs are derived from historical monthly climatology.
- Clear explanation that long-term climate trajectories and extreme anomaly dynamics are not modeled.
- Explicit statement that uncertainty may increase with projection horizon due to recursive error propagation and reliance on climatological predictor assumptions.
- Revised **Section 2.5.6 (Preparation of Future Predictor Variables)**.
- Revised **Section 2.6 (Model Validation and Robustness Assessment).**
- Addition of new Results subsection: **“3.3 Scenario-Based Projections with Uncertainty (2024–2027)”.**

Figure 8 was redesigned to:

- Clearly separate observed (2022–2023) and projection (2024–2027) periods using a vertical dashed boundary.
- Display time-varying empirical prediction intervals.

Furthermore, the discussion surrounding **Figure 9** has been refined to explicitly present the projected HI category distributions under a **“moderate scenario assumption”.** This ensures full transparency regarding scenario interpretation and prevents overinterpretation of long-term trajectories.

6. Data Availability and Transparency

Comment:

Clarification was requested regarding accessibility of processed datasets and code.

Response:

The Data Availability statement was updated to include:

- A public GitHub repository containing processed datasets and all Python scripts used for preprocessing, modeling, and scenario projections.
- Clarification that raw BMD meteorological data are subject to institutional policy but reproducible using the provided scripts.

This ensures compliance with journal transparency requirements.

Concluding Statement

We are deeply grateful for the reviewer's insightful comments, which significantly strengthened the manuscript. The revised version now:

- Clearly distinguishes conditional estimation from independent forecasting.
- Explicitly defines scenario-based projections.
- Rigorously addresses data leakage and validation.
- Incorporates baseline benchmarking.
- Provides transparent uncertainty quantification.
- Includes revised figures and a dedicated scenario-projection results section.
- Ensures full data and code transparency.

We believe the manuscript now presents a conceptually precise, statistically robust, and policy-relevant framework for high-resolution heat-stress assessment and anticipatory public health planning. Thank you again for your careful review and valuable guidance.

Sincerely,

Authors
