# Supplementary material for: Machine-learning framework for conditional estimation and scenario-based projection of the heat index for public health interventions
Source: PLoS One. 2026 Apr 28;21(4):e0348202. doi: 10.1371/journal.pone.0348202 (PMC13123980; doi:10.1371/journal.pone.0348202)
Supplement: Attachment — Submitted filename: Response_to_Reviewer_05.pdf [file pone.0348202.s006.pdf]

March 25, 2026

Machine-Learning Framework for Conditional Estimation and Scenario-Based Projection of the Heat Index for Public Health Interventions (Manuscript ID: PONE-D-25-44349R4)

Dear Editor,

We sincerely thank you and the reviewer for the careful evaluation of our manuscript and for the constructive comments, which have substantially improved the clarity, methodological rigor, and transparency of our work. We have carefully revised the manuscript to address all concerns. Below, we provide a detailed point-by-point response outlining how each issue has been addressed in the revised version.

Kind regards,
Authors

Journal Requirements

1. If the reviewer comments include a recommendation to cite specific previously published works, please review and evaluate these publications to determine whether they are relevant and should be cited. There is no requirement to cite these works unless the editor has indicated otherwise.

Author's Response:

The reviewer did not recommend any specific publications for citation. Therefore, no additional references were added.

2. Please review your reference list to ensure that it is complete and correct. If you have cited papers that have been retracted, please include the rationale for doing so in the manuscript text, or remove these references and replace them with relevant current references. Any changes to the reference list should be mentioned in the rebuttal letter that accompanies your revised manuscript. If you need to cite a retracted article, indicate the article's retracted status in the References list and also include a citation and full reference for the retraction notice.

Author's Response:

No issues with retracted or missing references were identified, and the reviewer did not suggest any additional references. Therefore, no changes were made to the reference list.

Reviewer #1 Comments

The revised manuscript provides a concise and pertinent examination of the estimation and projection of the Heat Index using machine-learning methods, with a particular emphasis on the heat risk in Dhaka. The study's strength is the comparison of multiple models using high-frequency meteorological data, which is crucial for urban public-health planning. In comparison to previous

revisions, the manuscript has been enhanced, particularly in the clarification of the modelling framework and the distinction between scenario-based analysis and short-term prediction analyses. Nevertheless, a few points still necessitate minor clarification. The Heat Index is derived from temperature and relative humidity, and as such, the manuscript should provide a more detailed explanation of the modelling approach's contribution. It should guarantee that conclusions are formulated with care. Furthermore, providing more detailed information regarding model validation, forecasting horizons, and uncertainty in projections would enhance methodological transparency. Minor language polishing would also enhance the readability of certain sections.

In conclusion, the investigation addresses a critical matter and offers valuable insights for the evaluation of heat risk. The manuscript would be suitable for publication with minor clarifications and enhancements to its presentation.

Author's Response:
We break the whole comments into points to provide a clear answer to each of them. The point-by-point responses are given below.

1. Clarification of the modelling contribution given that the Heat Index is deterministic.
Comment:
The Heat Index is derived from temperature and relative humidity, and as such, the manuscript should provide a more detailed explanation of the modelling approach's contribution.

Response:
We thank the reviewer for this important observation. To clarify the contribution of the proposed framework, we have explicitly emphasized throughout the manuscript (Abstract, Introduction, and Discussion) that the Heat Index (HI) is a deterministic function of air temperature and relative humidity. Accordingly, our framework does not aim to independently forecast HI, but instead performs conditional estimation based on meteorological predictors.

We have further strengthened this clarification by adding explicit statements highlighting that the key contribution of this study lies in capturing the nonlinear temporal dynamics, persistence, and high-frequency variability of HI using machine-learning approaches. This enables the transformation of meteorological inputs into operationally relevant, sub-daily heat-risk information, which is not directly obtainable from the static HI formulation. These revisions clearly distinguish the methodological contribution from the deterministic nature of HI and prevent misinterpretation as an independent forecasting system.

2. Ensuring conclusions are formulated with appropriate caution.

Comment:

It should guarantee that conclusions are formulated with care.

Response:

We have carefully revised the manuscript to ensure that all conclusions are stated with appropriate caution. Specifically, we consistently describe the results as conditional estimates and scenario-based projections, rather than deterministic forecasts. We also explicitly state that the proposed framework should complement rather than replace traditional meteorological forecasting systems, and that the projections depend on assumed meteorological inputs. These clarifications have been incorporated throughout the Abstract, Discussion, and Conclusion sections.

3. Additional detail on model validation, forecasting horizon, and uncertainty.

Comment:

Furthermore, providing more detailed information regarding model validation, forecasting horizons, and uncertainty in projections would enhance methodological transparency.

Response:

We thank the reviewer for this important suggestion. We have enhanced methodological transparency by adding explicit clarification statements within the relevant sections to better articulate the modeling framework.

- **Forecasting horizon:** We clarified that the framework operates as a 3-hour-ahead recursive conditional estimation system, and explicitly stated that longer-term outputs (2024–2027) represent scenario-based projections rather than direct long-horizon forecasts.
- **Scenario interpretation:** We added statements emphasizing that projections should not be interpreted as deterministic climate forecasts, but rather as conditional scenario analyses based on assumed meteorological inputs.
- **Uncertainty quantification:** We further clarified that the empirical tree-based prediction intervals reflect conditional model uncertainty under fixed inputs and do not capture the full uncertainty associated with future climate variability or extreme events.
- **Model validation:** We added a clarifying statement highlighting that the validation procedures (including expanding-window cross-validation and residual diagnostics) ensure robust generalization under temporally ordered data while minimizing overfitting and information leakage.

These additions improve clarity without altering the underlying methodology.

4. Minor language polishing

Comment:

Minor language polishing would also enhance the readability of certain sections.

Response:

We have carefully reviewed the manuscript and performed minor language refinements to improve clarity and readability, including simplifying long sentences and improving phrasing where necessary.
