# Supplementary material for: Machine-learning framework for conditional estimation and scenario-based projection of the heat index for public health interventions
Source: PLoS One. 2026 Apr 28;21(4):e0348202. doi: 10.1371/journal.pone.0348202 (PMC13123980; doi:10.1371/journal.pone.0348202)
Supplement: Peer review history

# Peer review: Decision Letter 0

**DOI:** 10.1371/journal.pone.0348202.r001

**Authors:** Lingye Yao

25 Sep 2025

Dear Dr. Binte Ahmed,

Please submit your revised manuscript by Nov 09 2025 11:59PM. If you will need more time than this to complete your revisions, please reply to this message or contact the journal office at plosone@plos.org. When you're ready to submit your revision, log on to https://www.editorialmanager.com/pone/ and select the 'Submissions Needing Revision' folder to locate your manuscript file.. When you're ready to submit your revision, log on to https://www.editorialmanager.com/pone/ and select the 'Submissions Needing Revision' folder to locate your manuscript file.. When you're ready to submit your revision, log on to https://www.editorialmanager.com/pone/ and select the 'Submissions Needing Revision' folder to locate your manuscript file.. When you're ready to submit your revision, log on to https://www.editorialmanager.com/pone/ and select the 'Submissions Needing Revision' folder to locate your manuscript file.

- A rebuttal letter that responds to each point raised by the academic editor and reviewer(s). You should upload this letter as a separate file labeled 'Response to Reviewers'.
- A marked-up copy of your manuscript that highlights changes made to the original version. You should upload this as a separate file labeled 'Revised Manuscript with Track Changes'.
- An unmarked version of your revised paper without tracked changes. You should upload this as a separate file labeled 'Manuscript'.

If you would like to make changes to your financial disclosure, please include your updated statement in your cover letter. Guidelines for resubmitting your figure files are available below the reviewer comments at the end of this letter.

If applicable, we recommend that you deposit your laboratory protocols in protocols.io to enhance the reproducibility of your results. Protocols.io assigns your protocol its own identifier (DOI) so that it can be cited independently in the future. For instructions see: https://journals.plos.org/plosone/s/submission-guidelines#loc-laboratory-protocols. Additionally, PLOS ONE offers an option for publishing peer-reviewed Lab Protocol articles, which describe protocols hosted on protocols.io. Read more information on sharing protocols at . Additionally, PLOS ONE offers an option for publishing peer-reviewed Lab Protocol articles, which describe protocols hosted on protocols.io. Read more information on sharing protocols at . Additionally, PLOS ONE offers an option for publishing peer-reviewed Lab Protocol articles, which describe protocols hosted on protocols.io. Read more information on sharing protocols at . Additionally, PLOS ONE offers an option for publishing peer-reviewed Lab Protocol articles, which describe protocols hosted on protocols.io. Read more information on sharing protocols at https://plos.org/protocols?utm_medium=editorial-email&utm_source=authorletters&utm_campaign=protocols....

We look forward to receiving your revised manuscript.

Kind regards,

Lingye Yao, Ph.D.

Academic Editor

PLOS ONE

**Journal Requirements:**

1. When submitting your revision, we need you to address these additional requirements.

Please ensure that your manuscript meets PLOS ONE's style requirements, including those for file naming. The PLOS ONE style templates can be found at

https://journals.plos.org/plosone/s/file?id=wjVg/PLOSOne_formatting_sample_main_body.pdf and

https://journals.plos.org/plosone/s/file?id=ba62/PLOSOne_formatting_sample_title_authors_affiliations.pdf

2. Please note that PLOS One has specific guidelines on code sharing for submissions in which author-generated code underpins the findings in the manuscript. In these cases, we expect all author-generated code to be made available without restrictions upon publication of the work. Please review our guidelines at https://journals.plos.org/plosone/s/materials-and-software-sharing#loc-sharing-code and ensure that your code is shared in a way that follows best practice and facilitates reproducibility and reuse.

3. Please include captions for your Supporting Information files at the end of your manuscript, and update any in-text citations to match accordingly. Please see our Supporting Information guidelines for more information: http://journals.plos.org/plosone/s/supporting-information.

4. If the reviewer comments include a recommendation to cite specific previously published works, please review and evaluate these publications to determine whether they are relevant and should be cited. There is no requirement to cite these works unless the editor has indicated otherwise. 

[Note: HTML markup is below. Please do not edit.]

Reviewers' comments:

**Comments to the Author**

1. Is the manuscript technically sound, and do the data support the conclusions?

Reviewer #1: Yes

Reviewer #2: Partly

**********

2. Has the statistical analysis been performed appropriately and rigorously?

Reviewer #1: Yes

Reviewer #2: No

**********

3. Have the authors made all data underlying the findings in their manuscript fully available?

The PLOS Data policy requires authors to make all data underlying the findings described in their manuscript fully available without restriction, with rare exception (please refer to the Data Availability Statement in the manuscript PDF file). The data should be provided as part of the manuscript or its supporting information, or deposited to a public repository. For example, in addition to summary statistics, the data points behind means, medians and variance measures should be available. If there are restrictions on publicly sharing data—e.g. participant privacy or use of data from a third party—those must be specified.requires authors to make all data underlying the findings described in their manuscript fully available without restriction, with rare exception (please refer to the Data Availability Statement in the manuscript PDF file). The data should be provided as part of the manuscript or its supporting information, or deposited to a public repository. For example, in addition to summary statistics, the data points behind means, medians and variance measures should be available. If there are restrictions on publicly sharing data—e.g. participant privacy or use of data from a third party—those must be specified.

Reviewer #1: Yes

Reviewer #2: Yes

**********

4. Is the manuscript presented in an intelligible fashion and written in standard English?

Reviewer #1: Yes

Reviewer #2: Yes

**********

**Reviewer #1:** This manuscript examines a critical and globally pertinent issue: the enhancement of urban waste management through the integration of AI and IoT systems. The theme is pertinent and strongly aligns with the increasing advocacy for intelligent, sustainable urban environments. I value how the paper frames waste management as not merely a logistical challenge but as a fundamental sustainability issue that intersects with urban health, environmental protection, and effective governance. The aspiration to incorporate AI and IoT into this discourse is laudable, and the prospective influence of these technologies is indisputable; instances like Barcelona’s intelligent waste bins and Seoul’s RFID-enabled food waste monitoring system demonstrate how data-centric systems can diminish operational expenses and enhance recycling rates. This manuscript examines a critical and globally pertinent issue: the enhancement of urban waste management through the integration of AI and IoT systems. The theme is pertinent and strongly aligns with the increasing advocacy for intelligent, sustainable urban environments. I value how the paper frames waste management as not merely a logistical challenge but as a fundamental sustainability issue that intersects with urban health, environmental protection, and effective governance. The aspiration to incorporate AI and IoT into this discourse is laudable, and the prospective influence of these technologies is indisputable; instances like Barcelona’s intelligent waste bins and Seoul’s RFID-enabled food waste monitoring system demonstrate how data-centric systems can diminish operational expenses and enhance recycling rates. This manuscript examines a critical and globally pertinent issue: the enhancement of urban waste management through the integration of AI and IoT systems. The theme is pertinent and strongly aligns with the increasing advocacy for intelligent, sustainable urban environments. I value how the paper frames waste management as not merely a logistical challenge but as a fundamental sustainability issue that intersects with urban health, environmental protection, and effective governance. The aspiration to incorporate AI and IoT into this discourse is laudable, and the prospective influence of these technologies is indisputable; instances like Barcelona’s intelligent waste bins and Seoul’s RFID-enabled food waste monitoring system demonstrate how data-centric systems can diminish operational expenses and enhance recycling rates. This manuscript examines a critical and globally pertinent issue: the enhancement of urban waste management through the integration of AI and IoT systems. The theme is pertinent and strongly aligns with the increasing advocacy for intelligent, sustainable urban environments. I value how the paper frames waste management as not merely a logistical challenge but as a fundamental sustainability issue that intersects with urban health, environmental protection, and effective governance. The aspiration to incorporate AI and IoT into this discourse is laudable, and the prospective influence of these technologies is indisputable; instances like Barcelona’s intelligent waste bins and Seoul’s RFID-enabled food waste monitoring system demonstrate how data-centric systems can diminish operational expenses and enhance recycling rates.

Nonetheless, I observed that the manuscript resembles a conceptual overview rather than a methodically organised research paper. The originality of the work remains ambiguous: does it present a novel framework, enhance existing AI-IoT models, or primarily constitute a literature synthesis? For instance, the paper references the predictive monitoring of waste levels via IoT sensors, a concept well-documented in previous research (e.g., experiments utilising ultrasonic sensor-based smart bins in Singapore). Your study must clearly articulate its unique perspective, whether by introducing an enhanced algorithm, examining a novel city-scale case study, or providing a comparative analysis of models.

The methodology section, in particular, requires significantly enhanced clarity. Currently, it is unclear whether the work relies on simulations, case studies, or an analysis of existing implementations. For example, if you assert efficiency improvements, are these derived from machine learning simulations (such as a neural network forecasting waste generation patterns) or based on pilot project data? Elucidating the selection of algorithms, be it support vector machines, random forests, or reinforcement learning, and their interaction with IoT systems is essential for readers to evaluate the technical integrity of the work. The absence of transparency restricts the study's reproducibility.

Likewise, the findings are presently articulated in general, nearly aspirational language. Assertions like “AI-driven IoT can enhance sustainability outcomes” are persuasive in theory but necessitate empirical validation. Consider incorporating case-based data: for instance, research in Sweden indicated that AI-assisted routing for waste trucks decreased fuel consumption by nearly 20%, while pilot projects in India revealed that real-time bin monitoring enhanced collection efficiency in densely populated informal settlements. Incorporating comparative evidence would enhance your argument and underscore the practical viability of your proposals.

The implications for policy and practice warrant greater emphasis. Municipalities, particularly in low- and middle-income nations, encounter obstacles in implementing AI-IoT systems, including substantial initial costs, disjointed governance, and restricted technical capabilities. Elaborating on the phased implementation of your model, beginning with high-density business districts and subsequently expanding to encompass entire cities, would enhance its practical relevance. Furthermore, sustainability encompasses not only efficiency but also equity. In what ways could marginalised neighbourhoods gain advantages from or be marginalised by these technologies? Incorporating this dimension would enhance the paper's social significance.

From a presentation standpoint, the manuscript is comprehensible but requires refinement. Numerous sections employ lengthy, redundant sentences, where more succinct phrasing would enhance coherence. Incorporating a schematic diagram of the proposed AI-IoT architecture would enhance readers' comprehension of the interactions among sensors, data analytics, and decision-making within the system. Likewise, tables that encapsulate current global implementations of smart waste initiatives could contextualise your research within the wider domain.

**Reviewer #2:** The manuscript addresses an important public health and climate adaptation challenge: forecasting the Heat Index (HI) for Dhaka using machine learning and statistical models. The topic is timely and relevant considering the increasing frequency of heatwaves in South Asia. The paper is well-written and organized, but various methodological problems need to be fixed and clarified before it can be published.The manuscript addresses an important public health and climate adaptation challenge: forecasting the Heat Index (HI) for Dhaka using machine learning and statistical models. The topic is timely and relevant considering the increasing frequency of heatwaves in South Asia. The paper is well-written and organized, but various methodological problems need to be fixed and clarified before it can be published.The manuscript addresses an important public health and climate adaptation challenge: forecasting the Heat Index (HI) for Dhaka using machine learning and statistical models. The topic is timely and relevant considering the increasing frequency of heatwaves in South Asia. The paper is well-written and organized, but various methodological problems need to be fixed and clarified before it can be published.The manuscript addresses an important public health and climate adaptation challenge: forecasting the Heat Index (HI) for Dhaka using machine learning and statistical models. The topic is timely and relevant considering the increasing frequency of heatwaves in South Asia. The paper is well-written and organized, but various methodological problems need to be fixed and clarified before it can be published.

Abstract:

- The abstract reports exact evaluation metrics (R², MAE, RMSE, etc.), which is too detailed for this section. Please highlight relative performance instead (e.g., “Random Forest outperformed other models”).

- How exactly did you forecast to 2027? What data did you use for future temperature and humidity? This isn't clear and needs explanation.

- The policy and public health implications are appropriate to include, but the language should be softened to avoid overstatement. Consider phrases like this “This framework could potentially support early warnings …”.

Introduction:

- You repeat some points (e.g., reliance on temperature-only warnings; global increase in heatwave days).

Methods:

- Please clarify if any gaps existed in the dataset and how they were handled; were they excluded, interpolated, etc.?

- Using a random 80/20 split for time series data creates data leakage - the model sees future patterns when training. You need to split by time periods instead.

- The future forecasting (2024–2027) methodology is fundamentally unclear. RF and XGBoost require exogenous predictors (temperature, humidity) that don't exist for future years. How were these obtained? If you only used seasonal patterns, say so and explain why that limits how useful the forecasts are.

Results:

- The Random Forest results (R² = 0.9860, MAPE = 0.02%) are unusually high for environmental forecasting. This suggests overfitting or that your validation method has problems.

- MAPE values of 0.02-0.08% seem artificially low. With high heat index values, percentage errors naturally look small even when absolute errors aren't. Consider more robust error metrics.

Discussion:

- Keep the Discussion focused on interpreting results and move broader implications, limitations, and future research into a separate section (e.g. “Conclusion”)

**********

what does this mean?). If published, this will include your full peer review and any attached files.). If published, this will include your full peer review and any attached files.). If published, this will include your full peer review and any attached files.). If published, this will include your full peer review and any attached files.

If you choose “no”, your identity will remain anonymous but your review may still be made public.

**Do you want your identity to be public for this peer review?** For information about this choice, including consent withdrawal, please see our For information about this choice, including consent withdrawal, please see our For information about this choice, including consent withdrawal, please see our For information about this choice, including consent withdrawal, please see our Privacy Policy..

Reviewer #1: **Yes:** SAYON PRAMANIKSAYON PRAMANIKSAYON PRAMANIKSAYON PRAMANIK

Reviewer #2: No

**********

[NOTE: If reviewer comments were submitted as an attachment file, they will be attached to this email and accessible via the submission site. Please log into your account, locate the manuscript record, and check for the action link "View Attachments". If this link does not appear, there are no attachment files.]

While revising your submission, please upload your figure files to the Preflight Analysis and Conversion Engine (PACE) digital diagnostic tool, https://pacev2.apexcovantage.com/. PACE helps ensure that figures meet PLOS requirements. To use PACE, you must first register as a user. Registration is free. Then, login and navigate to the UPLOAD tab, where you will find detailed instructions on how to use the tool. If you encounter any issues or have any questions when using PACE, please email PLOS at . PACE helps ensure that figures meet PLOS requirements. To use PACE, you must first register as a user. Registration is free. Then, login and navigate to the UPLOAD tab, where you will find detailed instructions on how to use the tool. If you encounter any issues or have any questions when using PACE, please email PLOS at . PACE helps ensure that figures meet PLOS requirements. To use PACE, you must first register as a user. Registration is free. Then, login and navigate to the UPLOAD tab, where you will find detailed instructions on how to use the tool. If you encounter any issues or have any questions when using PACE, please email PLOS at . PACE helps ensure that figures meet PLOS requirements. To use PACE, you must first register as a user. Registration is free. Then, login and navigate to the UPLOAD tab, where you will find detailed instructions on how to use the tool. If you encounter any issues or have any questions when using PACE, please email PLOS at figures@plos.org. Please note that Supporting Information files do not need this step.. Please note that Supporting Information files do not need this step.. Please note that Supporting Information files do not need this step.. Please note that Supporting Information files do not need this step.

# Author comment: Author response to Decision Letter 1

**DOI:** 10.1371/journal.pone.0348202.r002

24 Oct 2025

24th October, 2025

Forecasting Heat Index for Proactive Public Health Interventions: A Machine Learning Framework with Exogenous Predictors (Manuscript ID: PONE-D-25-44349)

Dear Editor,

We sincerely thank you and the reviewers for your time and thoughtful comments on our manuscript. We appreciate the opportunity to revise our work and have carefully addressed each concern to enhance the scientific quality, clarity, and structure of the manuscript. Below, we provide a detailed, point-by-point response to all the editorial and reviewer comments.

Best regards,

Authors

Editorial Requirements

1. When submitting your revision, we need you to address these additional requirements.

Please ensure that your manuscript meets PLOS ONE's style requirements, including those for file naming. The PLOS ONE style templates can be found at

https://journals.plos.org/plosone/s/file?id=wjVg/PLOSOne_formatting_sample_main_body.pdf and https://journals.plos.org/plosone/s/file?id=ba62/PLOSOne_formatting_sample_title_authors_affiliations.pdf

Author’s response:

We have carefully reviewed the PLOS ONE style templates and updated the manuscript to comply with all formatting requirements, including file naming conventions. All sections, headings, references, and figures now adhere to the journal’s style guidelines.

2. Please note that PLOS One has specific guidelines on code sharing for submissions in which author-generated code underpins the findings in the manuscript. In these cases, we expect all author-generated code to be made available without restrictions upon publication of the work. Please review our guidelines at

https://journals.plos.org/plosone/s/materials-and-software-sharing#loc-sharing-code and ensure that your code is shared in a way that follows best practice and facilitates reproducibility and reuse.

Author’s response:

We will share all author-generated code without restrictions in a public GitHub repository upon manuscript acceptance. The repository will be cited in the manuscript, include a detailed README file, and be archived on Zenodo to provide a permanent DOI. We confirm adherence to PLOS ONE's code sharing guidelines.

3. Please include captions for your Supporting Information files at the end of your manuscript, and update any in-text citations to match accordingly. Please see our Supporting Information guidelines for more information:

http://journals.plos.org/plosone/s/supporting-information.

Author’s response:

Captions for all Supporting Information files have been added at the end of the manuscript. All in-text references to Supporting Information have been updated to match these captions, in accordance with the journal’s guidelines.

4. If the reviewer comments include a recommendation to cite specific previously published works, please review and evaluate these publications to determine whether they are relevant and should be cited. There is no requirement to cite these works unless the editor has indicated otherwise.

Author’s response:

We have reviewed the publications recommended by the reviewers and evaluated their relevance to our study. Relevant works have been cited appropriately in the revised manuscript; citations unrelated to the study objectives have been omitted, consistent with the editor’s guidance.

Reviewer #1 Comments

This manuscript examines a critical and globally pertinent issue: the enhancement of urban waste management through the integration of AI and IoT systems. The theme is pertinent and strongly aligns with the increasing advocacy for intelligent, sustainable urban environments. I value how the paper frames waste management as not merely a logistical challenge but as a fundamental sustainability issue that intersects with urban health, environmental protection, and effective governance. The aspiration to incorporate AI and IoT into this discourse is laudable, and the prospective influence of these technologies is indisputable; instances like Barcelona’s intelligent waste bins and Seoul’s RFID-enabled food waste monitoring system demonstrate how data-centric systems can diminish operational expenses and enhance recycling rates.

Nonetheless, I observed that the manuscript resembles a conceptual overview rather than a methodically organized research paper. The originality of the work remains ambiguous: does it present a novel framework, enhance existing AI-IoT models, or primarily constitute a literature synthesis? For instance, the paper references the predictive monitoring of waste levels via IoT sensors, a concept well-documented in previous research (e.g., experiments utilizing ultrasonic sensor-based smart bins in Singapore). Your study must clearly articulate its unique perspective, whether by introducing an enhanced algorithm, examining a novel city-scale case study, or providing a comparative analysis of models.

The methodology section, in particular, requires significantly enhanced clarity. Currently, it is unclear whether the work relies on simulations, case studies, or an analysis of existing implementations. For example, if you assert efficiency improvements, are these derived from machine learning simulations (such as a neural network forecasting waste generation patterns) or based on pilot project data? Elucidating the selection of algorithms, be it support vector machines, random forests, or reinforcement learning, and their interaction with IoT systems is essential for readers to evaluate the technical integrity of the work. The absence of transparency restricts the study's reproducibility.

Likewise, the findings are presently articulated in general, nearly aspirational language. Assertions like “AI-driven IoT can enhance sustainability outcomes” are persuasive in theory but necessitate empirical validation. Consider incorporating case-based data: for instance, research in Sweden indicated that AI-assisted routing for waste trucks decreased fuel consumption by nearly 20%, while pilot projects in India revealed that real-time bin monitoring enhanced collection efficiency in densely populated informal settlements. Incorporating comparative evidence would enhance your argument and underscore the practical viability of your proposals.

The implications for policy and practice warrant greater emphasis. Municipalities, particularly in low- and middle-income nations, encounter obstacles in implementing AI-IoT systems, including substantial initial costs, disjointed governance, and restricted technical capabilities. Elaborating on the phased implementation of your model, beginning with high-density business districts and subsequently expanding to encompass entire cities, would enhance its practical relevance. Furthermore, sustainability encompasses not only efficiency but also equity. In what ways could marginalized neighborhoods gain advantages from or be marginalized by these technologies? Incorporating this dimension would enhance the paper's social significance.

From a presentation standpoint, the manuscript is comprehensible but requires refinement. Numerous sections employ lengthy, redundant sentences, where more succinct phrasing would enhance coherence. Incorporating a schematic diagram of the proposed AI-IoT architecture would enhance readers' comprehension of the interactions among sensors, data analytics, and decision-making within the system. Likewise, tables that encapsulate current global implementations of smart waste initiatives could contextualize your research within the wider domain.

Author’s response:

We sincerely thank Reviewer 1 for the time and effort devoted to reviewing our submission. However, upon careful reading, we believe that these comments may not relate to our manuscript entitled “Forecasting Heat Index for Proactive Public Health Interventions: A Machine Learning Framework with Exogenous Predictors.”

Reviewer 1’s feedback discusses AI- and IoT-based smart waste management systems, which appear to belong to another manuscript. Since our study focuses specifically on heat index forecasting using meteorological data (temperature, humidity, etc.), we could not identify a way to incorporate these comments without deviating from our research scope.

Accordingly, we respectfully note this possible mismatch for the editor’s attention. We remain committed to revising our manuscript in line with the relevant methodological and presentation concerns raised by Reviewer 2, and we have fully addressed those points in the revised version.

Reviewer #2 Comments

The manuscript addresses an important public health and climate adaptation challenge: forecasting the Heat Index (HI) for Dhaka using machine learning and statistical models. The topic is timely and relevant considering the increasing frequency of heatwaves in South Asia. The paper is well-written and organized, but various methodological problems need to be fixed and clarified before it can be published.

Abstract:

1. The abstract reports exact evaluation metrics (R², MAE, RMSE, etc.), which is too detailed for this section. Please highlight relative performance instead (e.g., “Random Forest outperformed other models”).

Author’s response:

We thank the reviewer for the suggestion. The abstract has been revised to focus on relative model performance rather than reporting exact numerical metrics. The Random Forest Regressor (RFR) is now described as achieving the highest predictive accuracy among all models, outperforming both statistical and deep learning approaches, while preserving clarity for the abstract section.

2. How exactly did you forecast to 2027? What data did you use for future temperature and humidity? This isn't clear and needs explanation.

Author’s response:

We thank the reviewer for this important observation. To forecast the Heat Index for 2024–2027, we used our trained Random Forest model, which requires temperature and humidity as input features. As actual meteorological data beyond 2023 were unavailable, we estimated future temperature and humidity using historical monthly averages from 2014–2023. These averages were repeated across the forecast horizon at 3-hour intervals to maintain seasonal and diurnal patterns. Small random variations were also added to temperature, humidity, and predicted Heat Index values to mimic real-world variability. This approach allowed the model to generate indicative forecasts reflecting typical seasonal dynamics rather than exact future values. The Abstract has been updated to briefly mention this procedure, and full methodological details can be found in Section 2.5.6 “Preparation of Future Predictor Variables for Forecasting” of the revised manuscript.

3. The policy and public health implications are appropriate to include, but the language should be softened to avoid overstatement. Consider phrases like this “This framework could potentially support early warnings …”.

Author’s response:

We thank the reviewer for the suggestion. The conclusion in the abstract has been revised to use more measured language. Phrases such as “could potentially support early warning systems” and “guide adaptive urban heat management strategies” have been adopted to convey practical implications without overstating certainty.

Introduction:

1. You repeat some points (e.g., reliance on temperature-only warnings; global increase in heatwave days)

Author’s response:

We thank the reviewer for pointing this out. The introduction has been revised to remove redundancy, consolidating mentions of temperature-only warnings and global heatwave trends into single, clear passages. The revised text now presents a concise, coherent narrative from global to local context, highlighting the research gap and objectives without repeating points.

Methods:

1. Please clarify if any gaps existed in the dataset and how they were handled; were they excluded, interpolated, etc.?

Author’s response:

We have explicitly noted that data completeness was verified during preprocessing and no missing values were found after validation. A clarifying sentence was added in Section 2.2.

2. Using a random 80/20 split for time series data creates data leakage - the model sees future patterns when training. You need to split by time periods instead.

Author’s response:

We thank the reviewer for the comment. The statement in the manuscript describing an 80/20 split was a textual mistake. In the analysis, the dataset was correctly split chronologically by time periods, with data from 2014–2021 used for training and 2022–2023 for testing. The manuscript has been corrected to reflect this in Section 2.4.

3. The future forecasting (2024–2027) methodology is fundamentally unclear. RF and XGBoost require exogenous predictors (temperature, humidity) that don't exist for future years. How were these obtained? If you only used seasonal patterns, say so and explain why that limits how useful the forecasts are.

Author’s response:

We thank the reviewer for this important observation. To clarify, we have added a new subsection in the Materials and Methods titled “2.5.6 Preparation of Future Predictor Variables for Forecasting”, which details how future exogenous variables were generated for all forecasting models. Specifically, temperature and humidity for 2024–2027 were approximated using historical monthly averages from 2014–2023, which were repeated at 3-hourly intervals to preserve seasonal and diurnal patterns. For machine learning models such as Random Forest, XGBoost, and LSTM, small random variations were introduced to mimic realistic variability, while statistical models (ARIMAX and SARIMAX) used the deterministic monthly averages directly.

We note that this approach assumes that near-future climatic conditions will follow historical seasonal cycles. As a result, the forecasts should be interpreted as scenario-based projections rather than precise predictions, and they do not capture potential long-term climate changes or anomalies. This limitation is now explicitly stated in the subsection to ensure transparency regarding the scope and interpretability of the future forecasts.

Results:

1. The Random Forest results (R² = 0.9860, MAPE = 0.02%) are unusually high for environmental forecasting. This suggests overfitting or that your validation method has problems.

Author’s response:

We thank the reviewer for the comment. The previously reported metrics were a reporting mistake. The correct metrics for the Random Forest model on the chronological test set are:

� R² = 0.9872

� MAE = 0.5485

� RMSE = 0.8473

� MAPE = 1.71%

� MASE = 0.2285

The high R² reflects the inclusion of lag features (HeatIndex_lag_1, lag_3, lag_6) and time-based features (Hour, Day, Month, Season), which capture strong short-term autocorrelation and seasonal patterns in the Heat Index. The model was evaluated on a chronological test set, so no future information was used in training. We have clarified this in the revised manuscript to prevent confusion regarding overfitting.

2. MAPE values of 0.02-0.08% seem artificially low. With high heat index values, percentage errors naturally look small even when absolute errors aren't. Consider more robust error metrics.

Author’s response:

We thank the reviewer for highlighting this. The previously reported MAPE values were due to a reporting mistake; the correct MAPE is 1.71%. To provide a more robust evaluation of model performance, we have now included MASE (Mean Absolute Scaled Error), which was not part of the original manuscript. The metrics table has been fully updated to report MAE, RMSE, MAPE, R², and MASE. MASE compares the model’s prediction errors to a naive forecast, making it less sensitive to high Heat Index values and providing a clearer assessment of predictive accuracy. This addition ensures that absolute and relative errors are transparently reported.

Discussion:

1. Keep the Discussion focused on interpreting results and move broader implications, limitations, and future research into a separate section (e.g. “Conclusion”)

Author’s response:

We sincerely thank the reviewer for this valuable suggestion. In the revised manuscript, the Discussion section has been refined to focus exclusively on interpreting model results, comparing performance metrics, and linking findings with re

Attachment Submitted filename: Response to Reviewers.pdf

# Peer review: Decision Letter 1

**DOI:** 10.1371/journal.pone.0348202.r003

**Authors:** Lingye Yao

26 Nov 2025

Dear Dr. Binte Ahmed,

Please submit your revised manuscript by Jan 10 2026 11:59PM. If you will need more time than this to complete your revisions, please reply to this message or contact the journal office at plosone@plos.org. When you're ready to submit your revision, log on to https://www.editorialmanager.com/pone/ and select the 'Submissions Needing Revision' folder to locate your manuscript file.. When you're ready to submit your revision, log on to https://www.editorialmanager.com/pone/ and select the 'Submissions Needing Revision' folder to locate your manuscript file.. When you're ready to submit your revision, log on to https://www.editorialmanager.com/pone/ and select the 'Submissions Needing Revision' folder to locate your manuscript file.. When you're ready to submit your revision, log on to https://www.editorialmanager.com/pone/ and select the 'Submissions Needing Revision' folder to locate your manuscript file.

- A rebuttal letter that responds to each point raised by the academic editor and reviewer(s). You should upload this letter as a separate file labeled 'Response to Reviewers'.
- A marked-up copy of your manuscript that highlights changes made to the original version. You should upload this as a separate file labeled 'Revised Manuscript with Track Changes'.
- An unmarked version of your revised paper without tracked changes. You should upload this as a separate file labeled 'Manuscript'.

If you would like to make changes to your financial disclosure, please include your updated statement in your cover letter. Guidelines for resubmitting your figure files are available below the reviewer comments at the end of this letter.

If applicable, we recommend that you deposit your laboratory protocols in protocols.io to enhance the reproducibility of your results. Protocols.io assigns your protocol its own identifier (DOI) so that it can be cited independently in the future. For instructions see: https://journals.plos.org/plosone/s/submission-guidelines#loc-laboratory-protocols. Additionally, PLOS ONE offers an option for publishing peer-reviewed Lab Protocol articles, which describe protocols hosted on protocols.io. Read more information on sharing protocols at . Additionally, PLOS ONE offers an option for publishing peer-reviewed Lab Protocol articles, which describe protocols hosted on protocols.io. Read more information on sharing protocols at . Additionally, PLOS ONE offers an option for publishing peer-reviewed Lab Protocol articles, which describe protocols hosted on protocols.io. Read more information on sharing protocols at . Additionally, PLOS ONE offers an option for publishing peer-reviewed Lab Protocol articles, which describe protocols hosted on protocols.io. Read more information on sharing protocols at https://plos.org/protocols?utm_medium=editorial-email&utm_source=authorletters&utm_campaign=protocols....

We look forward to receiving your revised manuscript.

Kind regards,

Lingye Yao, Ph.D.

Academic Editor

PLOS ONE

Journal Requirements:

If the reviewer comments include a recommendation to cite specific previously published works, please review and evaluate these publications to determine whether they are relevant and should be cited. There is no requirement to cite these works unless the editor has indicated otherwise.

[Note: HTML markup is below. Please do not edit.]

Reviewers' comments:

Reviewer's Responses to Questions

**Comments to the Author**

Reviewer #1: All comments have been addressed

Reviewer #2: All comments have been addressed

**********

2. Is the manuscript technically sound, and do the data support the conclusions?

Reviewer #1: Partly

Reviewer #2: Yes

**********

3. Has the statistical analysis been performed appropriately and rigorously?

Reviewer #1: Yes

Reviewer #2: Yes

**********

4. Have the authors made all data underlying the findings in their manuscript fully available?

The PLOS Data policy requires authors to make all data underlying the findings described in their manuscript fully available without restriction, with rare exception (please refer to the Data Availability Statement in the manuscript PDF file). The data should be provided as part of the manuscript or its supporting information, or deposited to a public repository. For example, in addition to summary statistics, the data points behind means, medians and variance measures should be available. If there are restrictions on publicly sharing data—e.g. participant privacy or use of data from a third party—those must be specified.requires authors to make all data underlying the findings described in their manuscript fully available without restriction, with rare exception (please refer to the Data Availability Statement in the manuscript PDF file). The data should be provided as part of the manuscript or its supporting information, or deposited to a public repository. For example, in addition to summary statistics, the data points behind means, medians and variance measures should be available. If there are restrictions on publicly sharing data—e.g. participant privacy or use of data from a third party—those must be specified.

Reviewer #1: Yes

Reviewer #2: Yes

**********

5. Is the manuscript presented in an intelligible fashion and written in standard English?

Reviewer #1: Yes

Reviewer #2: Yes

**********

Reviewer #1: The study offers a valuable analysis of environmental and climatic interactions using remote sensing data and statistical methods, but it needs considerable enhancement before acceptance. Although the topic is timely and the datasets are suitable, the methodological details, such as data preprocessing, model validation, and statistical robustness, are insufficiently explained. The discussion often repeats results without deeper interpretation or connection to existing literature, and causal inferences are drawn without sufficient evidence. The manuscript also requires thorough language editing to improve clarity and readability. Overall, the research shows promise but must undergo significant revision to strengthen its scientific rigour, transparency, and presentation quality.

Reviewer #2: (No Response)

**********

what does this mean?). If published, this will include your full peer review and any attached files.). If published, this will include your full peer review and any attached files.). If published, this will include your full peer review and any attached files.). If published, this will include your full peer review and any attached files.

If you choose “no”, your identity will remain anonymous but your review may still be made public.

**Do you want your identity to be public for this peer review?** For information about this choice, including consent withdrawal, please see our For information about this choice, including consent withdrawal, please see our For information about this choice, including consent withdrawal, please see our For information about this choice, including consent withdrawal, please see our Privacy Policy..

Reviewer #1: **Yes:** SAYON PRAMANIKSAYON PRAMANIKSAYON PRAMANIKSAYON PRAMANIK

Reviewer #2: No

**********

[NOTE: If reviewer comments were submitted as an attachment file, they will be attached to this email and accessible via the submission site. Please log into your account, locate the manuscript record, and check for the action link "View Attachments". If this link does not appear, there are no attachment files.]

To ensure your figures meet our technical requirements, please review our figure guidelines: https://journals.plos.org/plosone/s/figures

You may also use PLOS’s free figure tool, NAAS, to help you prepare publication quality figures: https://journals.plos.org/plosone/s/figures#loc-tools-for-figure-preparation.

NAAS will assess whether your figures meet our technical requirements by comparing each figure against our figure specifications.

# Author comment: Author response to Decision Letter 2

**DOI:** 10.1371/journal.pone.0348202.r004

5 Dec 2025

4th December, 2025

Forecasting Heat Index for Proactive Public Health Interventions: A Machine Learning Framework with Exogenous Predictors (Manuscript ID: PONE-D-25-44349R1)

Dear Editor,

We sincerely thank you and the reviewers for your time and thoughtful comments on our manuscript. We appreciate the opportunity to revise our work and have carefully addressed each concern to enhance the scientific quality, clarity, and structure of the manuscript. Below, we provide a detailed, point-by-point response to all the editorial and reviewer comments.

Best regards,

Authors

Journal Requirements

If the reviewer comments include a recommendation to cite specific previously published works, please review and evaluate these publications to determine whether they are relevant and should be cited. There is no requirement to cite these works unless the editor has indicated otherwise.

Author’s Response:

While the reviewer did not suggest specific publications, we have added several relevant references in the Discussion section of the revised manuscript to provide additional context and support.

Reviewer #1 Comments

The study offers a valuable analysis of environmental and climatic interactions using remote sensing data and statistical methods, but it needs considerable enhancement before acceptance. Although the topic is timely and the datasets are suitable, the methodological details, such as data preprocessing, model validation, and statistical robustness, are insufficiently explained. The discussion often repeats results without deeper interpretation or connection to existing literature, and causal inferences are drawn without sufficient evidence. The manuscript also requires thorough language editing to improve clarity and readability. Overall, the research shows promise but must undergo significant revision to strengthen its scientific rigour, transparency, and presentation quality.

Author’s Response:

We break the whole comments into points to provide a clear answer to each of them. The point-by-point responses are given below.

1. “The methodological details, such as data preprocessing, model validation, and statistical robustness, are insufficiently explained.”

Response:

Thank you for pointing this out. We have substantially expanded the Methods section to enhance transparency and reproducibility. In particular:

� A comprehensive, step-by-step preprocessing workflow, covering format conversion, dataset merging, timestamp validation, missing-value assessment, and outlier verification has been added in Section 2.2.

� Detailed explanations of model validation procedures and statistical robustness checks have been incorporated into a newly developed Section 2.6.

These revisions greatly improve the methodological clarity and completeness of the manuscript.

2. “The discussion often repeats results without deeper interpretation or connection to existing literature, and causal inferences are drawn without sufficient evidence.”

Response:

We appreciate this insightful observation. The Discussion section has been thoroughly rewritten to:

� Provide deeper interpretation rather than repeating numerical findings

� Connect the results with relevant and recent literature on heatwave forecasting, machine-learning-based climate models, and urban heat stress

� Clarify why the RFR model performed best

� Discuss implications for public health risk, urban planning, and climate-adaptation strategies in Dhaka

� Remove speculative language and avoid unsupported causal interpretations

These changes enhance the scholarly depth, relevance, and analytical quality of the Discussion.

3. “The manuscript requires thorough language editing to improve clarity and readability.”

Response:

The entire manuscript has been carefully edited for coherence, academic tone, grammar, and clarity. Long sentences were shortened, redundancies removed, and transitions improved. The revised version adheres to scientific writing standards and improves overall readability. Changes applied throughout the manuscript.

Attachment Submitted filename: Response_to_Reviewers_R2.pdf

# Peer review: Decision Letter 2

**DOI:** 10.1371/journal.pone.0348202.r005

**Authors:** Lingye Yao

23 Dec 2025

Dear Dr. Binte Ahmed,

Please submit your revised manuscript by Feb 06 2026 11:59PM. If you will need more time than this to complete your revisions, please reply to this message or contact the journal office at plosone@plos.org. When you're ready to submit your revision, log on to https://www.editorialmanager.com/pone/ and select the 'Submissions Needing Revision' folder to locate your manuscript file.. When you're ready to submit your revision, log on to https://www.editorialmanager.com/pone/ and select the 'Submissions Needing Revision' folder to locate your manuscript file.. When you're ready to submit your revision, log on to https://www.editorialmanager.com/pone/ and select the 'Submissions Needing Revision' folder to locate your manuscript file.. When you're ready to submit your revision, log on to https://www.editorialmanager.com/pone/ and select the 'Submissions Needing Revision' folder to locate your manuscript file.

- A letter that responds to each point raised by the academic editor and reviewer(s). You should upload this letter as a separate file labeled 'Response to Reviewers'.
- A marked-up copy of your manuscript that highlights changes made to the original version. You should upload this as a separate file labeled 'Revised Manuscript with Track Changes'.
- An unmarked version of your revised paper without tracked changes. You should upload this as a separate file labeled 'Manuscript'.

If you would like to make changes to your financial disclosure, please include your updated statement in your cover letter. Guidelines for resubmitting your figure files are available below the reviewer comments at the end of this letter.

If applicable, we recommend that you deposit your laboratory protocols in protocols.io to enhance the reproducibility of your results. Protocols.io assigns your protocol its own identifier (DOI) so that it can be cited independently in the future. For instructions see: https://journals.plos.org/plosone/s/submission-guidelines#loc-laboratory-protocols. Additionally, PLOS ONE offers an option for publishing peer-reviewed Lab Protocol articles, which describe protocols hosted on protocols.io. Read more information on sharing protocols at . Additionally, PLOS ONE offers an option for publishing peer-reviewed Lab Protocol articles, which describe protocols hosted on protocols.io. Read more information on sharing protocols at . Additionally, PLOS ONE offers an option for publishing peer-reviewed Lab Protocol articles, which describe protocols hosted on protocols.io. Read more information on sharing protocols at . Additionally, PLOS ONE offers an option for publishing peer-reviewed Lab Protocol articles, which describe protocols hosted on protocols.io. Read more information on sharing protocols at https://plos.org/protocols?utm_medium=editorial-email&utm_source=authorletters&utm_campaign=protocols....

We look forward to receiving your revised manuscript.

Kind regards,

Lingye Yao, Ph.D.

Academic Editor

PLOS One

Journal Requirements:

If the reviewer comments include a recommendation to cite specific previously published works, please review and evaluate these publications to determine whether they are relevant and should be cited. There is no requirement to cite these works unless the editor has indicated otherwise.

[Note: HTML markup is below. Please do not edit.]

Reviewers' comments:

Reviewer's Responses to Questions

**Comments to the Author**

Reviewer #1: All comments have been addressed

Reviewer #2: All comments have been addressed

**********

2. Is the manuscript technically sound, and do the data support the conclusions?

Reviewer #1: Partly

Reviewer #2: Yes

**********

3. Has the statistical analysis been performed appropriately and rigorously?

Reviewer #1: No

Reviewer #2: Yes

**********

4. Have the authors made all data underlying the findings in their manuscript fully available?

The PLOS Data policy requires authors to make all data underlying the findings described in their manuscript fully available without restriction, with rare exception (please refer to the Data Availability Statement in the manuscript PDF file). The data should be provided as part of the manuscript or its supporting information, or deposited to a public repository. For example, in addition to summary statistics, the data points behind means, medians and variance measures should be available. If there are restrictions on publicly sharing data—e.g. participant privacy or use of data from a third party—those must be specified.requires authors to make all data underlying the findings described in their manuscript fully available without restriction, with rare exception (please refer to the Data Availability Statement in the manuscript PDF file). The data should be provided as part of the manuscript or its supporting information, or deposited to a public repository. For example, in addition to summary statistics, the data points behind means, medians and variance measures should be available. If there are restrictions on publicly sharing data—e.g. participant privacy or use of data from a third party—those must be specified.

Reviewer #1: No

Reviewer #2: Yes

**********

5. Is the manuscript presented in an intelligible fashion and written in standard English?

Reviewer #1: Yes

Reviewer #2: Yes

**********

Reviewer #1: The revised manuscript demonstrates notable improvement in comparison to its previous iteration, especially concerning the structure and elucidation of research objectives. The subject matter remains pertinent, and the datasets employed are suitable for the scope of the investigation. Nonetheless, several fundamental issues warrant further attention prior to the work's publication readiness.

Although the methodological framework is largely appropriate, key analytical procedures require more comprehensive justification. While statistical procedures and parameter selections are articulated with greater clarity than previously, validation processes, uncertainty analyses, and robustness assessments remain somewhat limited. Consequently, some conclusions are presented with a strength that may not be fully supported by the data. The discussion section has been expanded; however, it still predominantly relies on descriptive interpretation rather than providing deeper mechanistic explanations or comparisons with closely related studies.

The manuscript is comprehensible; however, the quality of English language usage necessitates further refinement. Recurring grammatical errors, lengthy sentences, and redundant phrases detract from clarity and readability. Figures and tables are informative but would benefit from more precise captions and a clearer connection to the analytical narrative.

In summary, the study possesses considerable potential, but additional revisions are imperative to enhance methodological rigor, improve interpretative clarity, and refine linguistic precision.

Reviewer #2: (No Response)

**********

what does this mean?). If published, this will include your full peer review and any attached files.). If published, this will include your full peer review and any attached files.). If published, this will include your full peer review and any attached files.). If published, this will include your full peer review and any attached files.

If you choose “no”, your identity will remain anonymous but your review may still be made public.

**Do you want your identity to be public for this peer review?** For information about this choice, including consent withdrawal, please see our For information about this choice, including consent withdrawal, please see our For information about this choice, including consent withdrawal, please see our For information about this choice, including consent withdrawal, please see our Privacy Policy..

Reviewer #1: **Yes:** SAYON PRAMANIKSAYON PRAMANIKSAYON PRAMANIKSAYON PRAMANIK

Reviewer #2: No

**********

[NOTE: If reviewer comments were submitted as an attachment file, they will be attached to this email and accessible via the submission site. Please log into your account, locate the manuscript record, and check for the action link "View Attachments". If this link does not appear, there are no attachment files.]

To ensure your figures meet our technical requirements, please review our figure guidelines: https://journals.plos.org/plosone/s/figures

You may also use PLOS’s free figure tool, NAAS, to help you prepare publication quality figures: https://journals.plos.org/plosone/s/figures#loc-tools-for-figure-preparation.

NAAS will assess whether your figures meet our technical requirements by comparing each figure against our figure specifications.

# Author comment: Author response to Decision Letter 3

**DOI:** 10.1371/journal.pone.0348202.r006

12 Jan 2026

January 11, 2026

Forecasting Heat Index for Proactive Public Health Interventions: A Machine Learning Framework with Exogenous Predictors (Manuscript ID: PONE-D-25-44349R2)

Dear Editor,

We sincerely thank you and the reviewers for their time, careful evaluation, and constructive comments on our manuscript. We appreciate the opportunity to revise our work and have carefully addressed all concerns to improve the methodological rigor, interpretative depth, clarity, and overall presentation of the study. Below, we provide a detailed point-by-point response to the reviewer’s comments, indicating how each issue has been addressed in the revised manuscript.

Kind regards,

Authors

Journal Requirements

If the reviewer comments include a recommendation to cite specific previously published works, please review and evaluate these publications to determine whether they are relevant and should be cited. There is no requirement to cite these works unless the editor has indicated otherwise.

Author’s Response:

No specific publications were recommended for citation by the reviewer. Therefore, no additional references were added.

Reviewer #1 Comments

The revised manuscript demonstrates notable improvement in comparison to its previous iteration, especially concerning the structure and elucidation of research objectives. The subject matter remains pertinent, and the datasets employed are suitable for the scope of the investigation. Nonetheless, several fundamental issues warrant further attention prior to the work's publication readiness.

Although the methodological framework is largely appropriate, key analytical procedures require more comprehensive justification. While statistical procedures and parameter selections are articulated with greater clarity than previously, validation processes, uncertainty analyses, and robustness assessments remain somewhat limited. Consequently, some conclusions are presented with a strength that may not be fully supported by the data. The discussion section has been expanded; however, it still predominantly relies on descriptive interpretation rather than providing deeper mechanistic explanations or comparisons with closely related studies.

The manuscript is comprehensible; however, the quality of English language usage necessitates further refinement. Recurring grammatical errors, lengthy sentences, and redundant phrases detract from clarity and readability. Figures and tables are informative but would benefit from more precise captions and a clearer connection to the analytical narrative.

In summary, the study possesses considerable potential, but additional revisions are imperative to enhance methodological rigor, improve interpretative clarity, and refine linguistic precision.

Author’s Response:

We break the whole comments into points to provide a clear answer to each of them. The point-by-point responses are given below.

1. “Although the methodological framework is largely appropriate, key analytical procedures require more comprehensive justification.”

Response:

We thank the reviewer for this insightful comment. In the revised manuscript, we have substantially strengthened the justification of the analytical framework. Specifically, Section 2.5 now clearly explains the rationale for selecting ARIMAX and SARIMAX as statistical benchmarks, Random Forest and XGBoost as nonlinear ensemble learners, and LSTM as a deep learning approach for sequential dependency modeling. The motivation for using high-frequency (3-hourly) data and the Heat Index as a physiologically meaningful outcome variable has also been expanded. These revisions clarify the methodological choices and their relevance to urban heat-stress forecasting.

2. “Validation processes, uncertainty analyses, and robustness assessments remain somewhat limited.”

Response:

We thank the reviewer for this important observation. While a validation section was included in the previous version of the manuscript, Section 2.6 (Model Validation and Robustness Assessment) has now been substantially strengthened and refined in direct response to this comment.

In the revised manuscript, we clarify and expand the validation framework by explicitly describing the use of five-fold time-series cross-validation with strict preservation of chronological order to prevent information leakage and ensure realistic forecasting evaluation. The procedures for hyperparameter optimization are now described more clearly, and the role of early stopping in improving generalization for deep learning models is explicitly stated.

For the statistical models (ARIMAX and SARIMAX), the description of residual diagnostics has been refined to clearly document the assessment of autocorrelation, serial dependence, heteroscedasticity, and distributional conformity using autocorrelation and partial autocorrelation functions, Durbin–Watson statistics, Breusch–Pagan tests, and Q–Q plots. The revised text explicitly notes that these diagnostics did not indicate substantial violations of modeling assumptions.

In addition, we now explicitly address forecast robustness and uncertainty, noting that model performance was stable across cross-validation folds and clarifying that future projections are scenario-based estimates derived from climatological averages. This revision strengthens transparency regarding uncertainty while avoiding overstatement of predictive certainty.

We believe these revisions substantially improve the rigor, clarity, and completeness of the validation and robustness assessment and directly address the reviewer’s concern.

3. “Some conclusions are presented with a strength that may not be fully supported by the data.”

Response:

We thank the reviewer for highlighting this concern. In response, we have carefully revised the language throughout the Results, Discussion, and Conclusions sections to ensure appropriate scientific caution. Forecasts for the 2024–2027 period are now explicitly described as scenario-based projections, derived from climatological averages rather than climate-change-driven simulations. Statements implying deterministic prediction have been removed or softened, and uncertainty with increasing lead time is clearly acknowledged. These revisions ensure that the strength of the conclusions is fully aligned with the supporting evidence.

4. “The discussion section remains largely descriptive and would benefit from deeper mechanistic explanations and comparison with related studies.”

Response:

We appreciate this valuable suggestion. The Discussion section has been substantially expanded to include mechanistic interpretations of model performance. In particular, we explain why Random Forest Regression outperformed other approaches by highlighting its ability to capture nonlinear temperature–humidity interactions and threshold effects inherent in Heat Index dynamics. The comparatively weaker performance of LSTM and XGBoost is discussed in the context of high-frequency, noise-dominated climatic time series.

We have also strengthened comparisons with closely related studies, including prior work on Heat Index forecasting and heatwave modeling in South Asia and comparable climates. These additions move the discussion beyond descriptive interpretation and provide a deeper process-based understanding of the results.

5. “The quality of English language usage requires further refinement, and figures and tables would benefit from more precise captions and clearer linkage to the analytical narrative.”

Response:

We thank the reviewer for this comment. The manuscript has undergone careful language polishing to improve clarity, conciseness, and readability. Long sentences have been shortened, redundant phrasing removed, and grammatical consistency improved throughout the text.

Figure and table captions have been revised to be more precise and informative, explicitly linking visual outputs to their analytical and public-health relevance. The revised captions now clearly describe the content, interpretation, and significance of each figure and table within the broader narrative.

Attachment Submitted filename: Response to Reviewers_03.pdf

# Peer review: Decision Letter 3

**DOI:** 10.1371/journal.pone.0348202.r007

**Authors:** Lingye Yao

5 Feb 2026

Dear Dr. Binte Ahmed,

Please submit your revised manuscript by Mar 22 2026 11:59PM. If you will need more time than this to complete your revisions, please reply to this message or contact the journal office at plosone@plos.org. When you're ready to submit your revision, log on to https://www.editorialmanager.com/pone/ and select the 'Submissions Needing Revision' folder to locate your manuscript file.. When you're ready to submit your revision, log on to https://www.editorialmanager.com/pone/ and select the 'Submissions Needing Revision' folder to locate your manuscript file.. When you're ready to submit your revision, log on to https://www.editorialmanager.com/pone/ and select the 'Submissions Needing Revision' folder to locate your manuscript file.. When you're ready to submit your revision, log on to https://www.editorialmanager.com/pone/ and select the 'Submissions Needing Revision' folder to locate your manuscript file.

- A letter that responds to each point raised by the academic editor and reviewer(s). You should upload this letter as a separate file labeled 'Response to Reviewers'.
- A marked-up copy of your manuscript that highlights changes made to the original version. You should upload this as a separate file labeled 'Revised Manuscript with Track Changes'.
- An unmarked version of your revised paper without tracked changes. You should upload this as a separate file labeled 'Manuscript'.

If you would like to make changes to your financial disclosure, please include your updated statement in your cover letter. Guidelines for resubmitting your figure files are available below the reviewer comments at the end of this letter.

If applicable, we recommend that you deposit your laboratory protocols in protocols.io to enhance the reproducibility of your results. Protocols.io assigns your protocol its own identifier (DOI) so that it can be cited independently in the future. For instructions see: https://journals.plos.org/plosone/s/submission-guidelines#loc-laboratory-protocols. Additionally, PLOS ONE offers an option for publishing peer-reviewed Lab Protocol articles, which describe protocols hosted on protocols.io. Read more information on sharing protocols at . Additionally, PLOS ONE offers an option for publishing peer-reviewed Lab Protocol articles, which describe protocols hosted on protocols.io. Read more information on sharing protocols at . Additionally, PLOS ONE offers an option for publishing peer-reviewed Lab Protocol articles, which describe protocols hosted on protocols.io. Read more information on sharing protocols at . Additionally, PLOS ONE offers an option for publishing peer-reviewed Lab Protocol articles, which describe protocols hosted on protocols.io. Read more information on sharing protocols at https://plos.org/protocols?utm_medium=editorial-email&utm_source=authorletters&utm_campaign=protocols....

We look forward to receiving your revised manuscript.

Kind regards,

Lingye Yao, Ph.D.

Academic Editor

PLOS One

Journal Requirements:

If the reviewer comments include a recommendation to cite specific previously published works, please review and evaluate these publications to determine whether they are relevant and should be cited. There is no requirement to cite these works unless the editor has indicated otherwise.

[Note: HTML markup is below. Please do not edit.]

Reviewers' comments:

Reviewer's Responses to Questions

**Comments to the Author**

Reviewer #1: All comments have been addressed

Reviewer #2: All comments have been addressed

**********

2. Is the manuscript technically sound, and do the data support the conclusions?

Reviewer #1: Partly

Reviewer #2: Yes

**********

3. Has the statistical analysis been performed appropriately and rigorously?

Reviewer #1: No

Reviewer #2: Yes

**********

4. Have the authors made all data underlying the findings in their manuscript fully available?

The PLOS Data policy requires authors to make all data underlying the findings described in their manuscript fully available without restriction, with rare exception (please refer to the Data Availability Statement in the manuscript PDF file). The data should be provided as part of the manuscript or its supporting information, or deposited to a public repository. For example, in addition to summary statistics, the data points behind means, medians and variance measures should be available. If there are restrictions on publicly sharing data—e.g. participant privacy or use of data from a third party—those must be specified.requires authors to make all data underlying the findings described in their manuscript fully available without restriction, with rare exception (please refer to the Data Availability Statement in the manuscript PDF file). The data should be provided as part of the manuscript or its supporting information, or deposited to a public repository. For example, in addition to summary statistics, the data points behind means, medians and variance measures should be available. If there are restrictions on publicly sharing data—e.g. participant privacy or use of data from a third party—those must be specified.

Reviewer #1: No

Reviewer #2: Yes

**********

5. Is the manuscript presented in an intelligible fashion and written in standard English?

Reviewer #1: Yes

Reviewer #2: Yes

**********

Reviewer #1: The manuscript addresses a significant and timely public health concern by examining the dynamics of the Heat Index through the utilization of high-frequency meteorological data and multiple modeling approaches. The overall motivation is well-articulated, and the comparative modeling efforts constitute a notable positive aspect. However, despite enhancements over earlier versions, several critical issues persist that undermine the manuscript’s technical robustness.

A primary concern pertains to the conceptual framing of the study. The Heat Index is a deterministic function of temperature and relative humidity; nevertheless, the manuscript continues to portray the task as “forecasting” the Heat Index without explicitly clarifying the operational assumptions involved. The contribution would be considerably strengthened if the authors explicitly contextualized the work as conditional Heat Index estimation based on assumed future meteorological inputs or, alternatively, concentrated on forecasting temperature and humidity prior to deriving the Heat Index.

Furthermore, the statistical rigor requires reinforcement. Key details regarding forecasting horizons, measures to prevent data leakage when utilizing lagged variables, comparisons with baseline models, and methods for quantifying uncertainty are either omitted or insufficiently addressed. The long-term analysis (2024–2027), which relies on historical averages supplemented with random perturbations, limits the model's capacity to accurately represent extreme events and diminishes the robustness of the quantitative conclusions derived from these scenarios.

In addition, data availability does not fully comply with publication guidelines, given that it is unclear whether all processed datasets and analysis codes are publicly accessible. In summary, while the study demonstrates considerable potential, it necessitates further revision to improve conceptual clarity, methodological rigor, data transparency, and presentation quality.

Reviewer #2: (No Response)

**********

what does this mean?). If published, this will include your full peer review and any attached files.). If published, this will include your full peer review and any attached files.). If published, this will include your full peer review and any attached files.). If published, this will include your full peer review and any attached files.

If you choose “no”, your identity will remain anonymous but your review may still be made public.

**Do you want your identity to be public for this peer review?** For information about this choice, including consent withdrawal, please see our For information about this choice, including consent withdrawal, please see our For information about this choice, including consent withdrawal, please see our For information about this choice, including consent withdrawal, please see our Privacy Policy..

Reviewer #1: **Yes:** SAYON PRAMANIKSAYON PRAMANIKSAYON PRAMANIKSAYON PRAMANIK

Reviewer #2: No

**********

[NOTE: If reviewer comments were submitted as an attachment file, they will be attached to this email and accessible via the submission site. Please log into your account, locate the manuscript record, and check for the action link "View Attachments". If this link does not appear, there are no attachment files.]

To ensure your figures meet our technical requirements, please review our figure guidelines: https://journals.plos.org/plosone/s/figures

You may also use PLOS’s free figure tool, NAAS, to help you prepare publication quality figures: https://journals.plos.org/plosone/s/figures#loc-tools-for-figure-preparation.

NAAS will assess whether your figures meet our technical requirements by comparing each figure against our figure specifications.

# Author comment: Author response to Decision Letter 4

**DOI:** 10.1371/journal.pone.0348202.r008

3 Mar 2026

March 04, 2026

Machine-Learning Framework for Conditional Estimation and Scenario-Based Projection of the Heat Index for Public Health Interventions (Manuscript ID: PONE-D-25-44349R3)

Dear Editor,

We sincerely thank you and the reviewer for the careful evaluation of our manuscript and for the constructive comments that have helped us substantially improve the conceptual clarity, methodological rigor, and transparency of our work. We have carefully revised the manuscript to address all concerns. Below, we provide a detailed point-by-point response outlining how each issue has been resolved in the revised version.

Kind regards,

Authors

Journal Requirements

If the reviewer comments include a recommendation to cite specific previously published works, please review and evaluate these publications to determine whether they are relevant and should be cited. There is no requirement to cite these works unless the editor has indicated otherwise.

Author’s Response:

No specific publications were recommended for citation by the reviewer. Therefore, no additional references were added.

Reviewer #1 Comments

The manuscript addresses a significant and timely public health concern by examining the dynamics of the Heat Index through the utilization of high-frequency meteorological data and multiple modeling approaches. The overall motivation is well-articulated, and the comparative modeling efforts constitute a notable positive aspect. However, despite enhancements over earlier versions, several critical issues persist that undermine the manuscript’s technical robustness.

A primary concern pertains to the conceptual framing of the study. The Heat Index is a deterministic function of temperature and relative humidity; nevertheless, the manuscript continues to portray the task as “forecasting” the Heat Index without explicitly clarifying the operational assumptions involved. The contribution would be considerably strengthened if the authors explicitly contextualized the work as conditional Heat Index estimation based on assumed future meteorological inputs or, alternatively, concentrated on forecasting temperature and humidity prior to deriving the Heat Index.

Furthermore, the statistical rigor requires reinforcement. Key details regarding forecasting horizons, measures to prevent data leakage when utilizing lagged variables, comparisons with baseline models, and methods for quantifying uncertainty are either omitted or insufficiently addressed. The long-term analysis (2024–2027), which relies on historical averages supplemented with random perturbations, limits the model's capacity to accurately represent extreme events and diminishes the robustness of the quantitative conclusions derived from these scenarios.

In addition, data availability does not fully comply with publication guidelines, given that it is unclear whether all processed datasets and analysis codes are publicly accessible. In summary, while the study demonstrates considerable potential, it necessitates further revision to improve conceptual clarity, methodological rigor, data transparency, and presentation quality.

Author’s Response:

We break the whole comments into points to provide a clear answer to each of them. The point-by-point responses are given below.

1. Conceptual Framing of Heat Index “Forecasting”

Comment:

The Heat Index (HI) is a deterministic function of temperature and relative humidity; therefore, portraying the task as “forecasting” HI requires clearer operational assumptions.

Response:

We fully agree and have substantially clarified the conceptual framing throughout the manuscript. The following revisions were implemented:

� The manuscript title has been revised from “Forecasting Heat Index for Proactive Public Health Interventions: A Machine Learning Framework with Exogenous Predictors” to “Machine-Learning Framework for Conditional Estimation and Scenario-Based Projection of the Heat Index for Public Health Interventions” to reflect the clarified conceptual framing.

� We explicitly state throughout the manuscript that HI is deterministically derived from air temperature and relative humidity.

� The modeling task is now consistently described as conditional estimation under assumed meteorological inputs rather than independent climate forecasting.

� The Discussion clarifies that the added value of the framework lies in capturing nonlinear temporal persistence and translating conditional meteorological scenarios into actionable sub-daily projections.

These changes ensure conceptual precision and prevent misinterpretation regarding deterministic properties of HI.

2. Forecasting Horizon and Prevention of Data Leakage

Comment:

Clarification was requested regarding forecasting horizons and safeguards against data leakage when using lagged variables.

Response:

We strengthened Sections 2.4, 2.5, and 2.6 to clarify:

� Training period: 2014–2021

� Test period: 2022–2023

� Recursive 3-hour-ahead conditional estimation framework

� Strict chronological partitioning

� Expanding-window five-fold time-series cross-validation

We explicitly state that:

� All lagged HI predictors were generated strictly from past observations within each temporal partition.

� Feature scaling was performed using training data only.

� No information from the test period was used during feature construction.

These revisions eliminate potential data leakage concerns.

3. Baseline Model Comparison

Comment:

Inclusion of a baseline model for comparison was recommended.

Response:

We incorporated a naïve persistence benchmark (HIₜ = HIₜ₋₁) and evaluated performance using MAE, RMSE, R², and MASE. The inclusion of MASE provides a scale-independent comparison relative to the naive baseline. All evaluated models achieved MASE < 1, confirming improved performance over the persistence benchmark. This strengthens the comparative rigor of the study.

4. Uncertainty Quantification

Comment:

Greater clarity was requested regarding uncertainty estimation.

Response:

We expanded uncertainty quantification by:

� Implementing empirical 95% prediction intervals derived from ensemble tree dispersion.

� Adding a new subsection: “2.5.7 Empirical Tree-Based Prediction Intervals”

� Reporting empirical coverage probability (98.85%) and average interval width (3.94°C).

� Adding an “Empirical Prediction Interval Performance” subsection in Results (Section 3.2).

� Revising Section 2.6 (Model Validation and Robustness Assessment).

We emphasize that prediction intervals quantify conditional model dispersion under fixed meteorological inputs and do not incorporate structural climate-model uncertainty. These revisions substantially improve statistical transparency.

5. Long-Term Projections (2024–2027)

Comment:

The long-term scenario construction based on historical averages may limit representation of extreme events.

Response:

We revised the projection framework to clearly define these outputs as scenario-based climatological projections, not climate-change forecasts.

Revisions include:

� Explicit clarification that future temperature and humidity inputs are derived from historical monthly climatology.

� Clear explanation that long-term climate trajectories and extreme anomaly dynamics are not modeled.

� Explicit statement that uncertainty may increase with projection horizon due to recursive error propagation and reliance on climatological predictor assumptions.

� Revised Section 2.5.6 (Preparation of Future Predictor Variables).

� Revised Section 2.6 (Model Validation and Robustness Assessment).

� Addition of new Results subsection: “3.3 Scenario-Based Projections with Uncertainty (2024–2027)”.

Figure 8 was redesigned to:

� Clearly separate observed (2022–2023) and projection (2024–2027) periods using a vertical dashed boundary.

� Display time-varying empirical prediction intervals.

Furthermore, the discussion surrounding Figure 9 has been refined to explicitly present the projected HI category distributions under a “moderate scenario assumption”. This ensures full transparency regarding scenario interpretation and prevents overinterpretation of long-term trajectories.

6. Data Availability and Transparency

Comment:

Clarification was requested regarding accessibility of processed datasets and code.

Response:

The Data Availability statement was updated to include:

� A public GitHub repository containing processed datasets and all Python scripts used for preprocessing, modeling, and scenario projections.

� Clarification that raw BMD meteorological data are subject to institutional policy but reproducible using the provided scripts.

This ensures compliance with journal transparency requirements.

Concluding Statement

We are deeply grateful for the reviewer’s insightful comments, which significantly strengthened the manuscript. The revised version now:

� Clearly distinguishes conditional estimation from independent forecasting.

� Explicitly defines scenario-based projections.

� Rigorously addresses data leakage and validation.

� Incorporates baseline benchmarking.

� Provides transparent uncertainty quantification.

� Includes revised figures and a dedicated scenario-projection results section.

� Ensures full data and code transparency.

We believe the manuscript now presents a conceptually precise, statistically robust, and policy-relevant framework for high-resolution heat-stress assessment and anticipatory public health planning. Thank you again for your careful review and valuable guidance.

Sincerely,

Authors

Attachment Submitted filename: Response_to_Reviewer_04.pdf

# Peer review: Decision Letter 4

**DOI:** 10.1371/journal.pone.0348202.r009

**Authors:** Lingye Yao

23 Mar 2026

Dear Dr. Binte Ahmed,

Thank you for submitting your manuscript to PLOS ONE. After careful consideration, we feel that it has merit but does not fully meet PLOS ONE’s publication criteria as it currently stands. Therefore, we invite you to submit a revised version of the manuscript that addresses the points raised during the review process.

==============================

Please submit your revised manuscript by May 07 2026 11:59PM. If you will need more time than this to complete your revisions, please reply to this message or contact the journal office at plosone@plos.org. When you're ready to submit your revision, log on to https://www.editorialmanager.com/pone/ and select the 'Submissions Needing Revision' folder to locate your manuscript file.. When you're ready to submit your revision, log on to https://www.editorialmanager.com/pone/ and select the 'Submissions Needing Revision' folder to locate your manuscript file.. When you're ready to submit your revision, log on to https://www.editorialmanager.com/pone/ and select the 'Submissions Needing Revision' folder to locate your manuscript file.. When you're ready to submit your revision, log on to https://www.editorialmanager.com/pone/ and select the 'Submissions Needing Revision' folder to locate your manuscript file.

- A letter that responds to each point raised by the academic editor and reviewer(s). You should upload this letter as a separate file labeled 'Response to Reviewers'.
- A marked-up copy of your manuscript that highlights changes made to the original version. You should upload this as a separate file labeled 'Revised Manuscript with Track Changes'.
- An unmarked version of your revised paper without tracked changes. You should upload this as a separate file labeled 'Manuscript'.

If applicable, we recommend that you deposit your laboratory protocols in protocols.io to enhance the reproducibility of your results. Protocols.io assigns your protocol its own identifier (DOI) so that it can be cited independently in the future. For instructions see: https://journals.plos.org/plosone/s/submission-guidelines#loc-laboratory-protocols. Additionally, PLOS ONE offers an option for publishing peer-reviewed Lab Protocol articles, which describe protocols hosted on protocols.io. Read more information on sharing protocols at . Additionally, PLOS ONE offers an option for publishing peer-reviewed Lab Protocol articles, which describe protocols hosted on protocols.io. Read more information on sharing protocols at . Additionally, PLOS ONE offers an option for publishing peer-reviewed Lab Protocol articles, which describe protocols hosted on protocols.io. Read more information on sharing protocols at . Additionally, PLOS ONE offers an option for publishing peer-reviewed Lab Protocol articles, which describe protocols hosted on protocols.io. Read more information on sharing protocols at https://plos.org/protocols?utm_medium=editorial-email&utm_source=authorletters&utm_campaign=protocols....

We look forward to receiving your revised manuscript.

Kind regards,

Lingye Yao, Ph.D.

Academic Editor

PLOS One

Journal Requirements:

1. If the reviewer comments include a recommendation to cite specific previously published works, please review and evaluate these publications to determine whether they are relevant and should be cited. There is no requirement to cite these works unless the editor has indicated otherwise.

2. Please review your reference list to ensure that it is complete and correct. If you have cited papers that have been retracted, please include the rationale for doing so in the manuscript text, or remove these references and replace them with relevant current references. Any changes to the reference list should be mentioned in the rebuttal letter that accompanies your revised manuscript. If you need to cite a retracted article, indicate the article’s retracted status in the References list and also include a citation and full reference for the retraction notice.

[Note: HTML markup is below. Please do not edit.]

Reviewers' comments:

Reviewer's Responses to Questions

**Comments to the Author**

Reviewer #1: All comments have been addressed

Reviewer #2: All comments have been addressed

**********

2. Is the manuscript technically sound, and do the data support the conclusions?

Reviewer #1: Yes

Reviewer #2: Yes

**********

3. Has the statistical analysis been performed appropriately and rigorously?

Reviewer #1: Yes

Reviewer #2: Yes

**********

4. Have the authors made all data underlying the findings in their manuscript fully available?

The PLOS Data policy requires authors to make all data underlying the findings described in their manuscript fully available without restriction, with rare exception (please refer to the Data Availability Statement in the manuscript PDF file). The data should be provided as part of the manuscript or its supporting information, or deposited to a public repository. For example, in addition to summary statistics, the data points behind means, medians and variance measures should be available. If there are restrictions on publicly sharing data—e.g. participant privacy or use of data from a third party—those must be specified.requires authors to make all data underlying the findings described in their manuscript fully available without restriction, with rare exception (please refer to the Data Availability Statement in the manuscript PDF file). The data should be provided as part of the manuscript or its supporting information, or deposited to a public repository. For example, in addition to summary statistics, the data points behind means, medians and variance measures should be available. If there are restrictions on publicly sharing data—e.g. participant privacy or use of data from a third party—those must be specified.

Reviewer #1: Yes

Reviewer #2: Yes

**********

5. Is the manuscript presented in an intelligible fashion and written in standard English?

Reviewer #1: Yes

Reviewer #2: Yes

**********

Reviewer #1: The revised manuscript provides a concise and pertinent examination of the estimation and projection of the Heat Index using machine-learning methods, with a particular emphasis on the heat risk in Dhaka. The study's strength is the comparison of multiple models using high-frequency meteorological data, which is crucial for urban public-health planning. In comparison to previous revisions, the manuscript has been enhanced, particularly in the clarification of the modelling framework and the distinction between scenario-based analysis and short-term prediction analyses.

Nevertheless, a few points still necessitate minor clarification. The Heat Index is derived from temperature and relative humidity, and as such, the manuscript should provide a more detailed explanation of the modelling approach's contribution. It should guarantee that conclusions are formulated with care. Furthermore, providing more detailed information regarding model validation, forecasting horizons, and uncertainty in projections would enhance methodological transparency. Minor language polishing would also enhance the readability of certain sections.

In conclusion, the investigation addresses a critical matter and offers valuable insights for the evaluation of heat risk. The manuscript would be suitable for publication with minor clarifications and enhancements to its presentation.

Reviewer #2: (No Response)

**********

what does this mean?). If published, this will include your full peer review and any attached files.). If published, this will include your full peer review and any attached files.). If published, this will include your full peer review and any attached files.). If published, this will include your full peer review and any attached files.

If you choose “no”, your identity will remain anonymous but your review may still be made public.

**Do you want your identity to be public for this peer review?** For information about this choice, including consent withdrawal, please see our For information about this choice, including consent withdrawal, please see our For information about this choice, including consent withdrawal, please see our For information about this choice, including consent withdrawal, please see our Privacy Policy..

Reviewer #1: **Yes:** SAYON PRAMANIKSAYON PRAMANIKSAYON PRAMANIKSAYON PRAMANIK

Reviewer #2: No

**********

[NOTE: If reviewer comments were submitted as an attachment file, they will be attached to this email and accessible via the submission site. Please log into your account, locate the manuscript record, and check for the action link "View Attachments". If this link does not appear, there are no attachment files.]

To ensure your figures meet our technical requirements, please review our figure guidelines: https://journals.plos.org/plosone/s/figures

You may also use PLOS’s free figure tool, NAAS, to help you prepare publication quality figures: https://journals.plos.org/plosone/s/figures#loc-tools-for-figure-preparation.

NAAS will assess whether your figures meet our technical requirements by comparing each figure against our figure specifications.

# Author comment: Author response to Decision Letter 5

**DOI:** 10.1371/journal.pone.0348202.r010

25 Mar 2026

March 25, 2026

Machine-Learning Framework for Conditional Estimation and Scenario-Based Projection of the Heat Index for Public Health Interventions (Manuscript ID: PONE-D-25-44349R4)

Dear Editor,

We sincerely thank you and the reviewer for the careful evaluation of our manuscript and for the constructive comments, which have substantially improved the clarity, methodological rigor, and transparency of our work. We have carefully revised the manuscript to address all concerns. Below, we provide a detailed point-by-point response outlining how each issue has been addressed in the revised version.

Kind regards,

Authors

Journal Requirements

1. If the reviewer comments include a recommendation to cite specific previously published works, please review and evaluate these publications to determine whether they are relevant and should be cited. There is no requirement to cite these works unless the editor has indicated otherwise.

Author’s Response:

The reviewer did not recommend any specific publications for citation. Therefore, no additional references were added.

2. Please review your reference list to ensure that it is complete and correct. If you have cited papers that have been retracted, please include the rationale for doing so in the manuscript text, or remove these references and replace them with relevant current references. Any changes to the reference list should be mentioned in the rebuttal letter that accompanies your revised manuscript. If you need to cite a retracted article, indicate the article’s retracted status in the References list and also include a citation and full reference for the retraction notice.

Author’s Response:

No issues with retracted or missing references were identified, and the reviewer did not suggest any additional references. Therefore, no changes were made to the reference list.

Reviewer #1 Comments

The revised manuscript provides a concise and pertinent examination of the estimation and projection of the Heat Index using machine-learning methods, with a particular emphasis on the heat risk in Dhaka. The study's strength is the comparison of multiple models using high-frequency meteorological data, which is crucial for urban public-health planning. In comparison to previous revisions, the manuscript has been enhanced, particularly in the clarification of the modelling framework and the distinction between scenario-based analysis and short-term prediction analyses. Nevertheless, a few points still necessitate minor clarification. The Heat Index is derived from temperature and relative humidity, and as such, the manuscript should provide a more detailed explanation of the modelling approach's contribution. It should guarantee that conclusions are formulated with care. Furthermore, providing more detailed information regarding model validation, forecasting horizons, and uncertainty in projections would enhance methodological transparency. Minor language polishing would also enhance the readability of certain sections.

In conclusion, the investigation addresses a critical matter and offers valuable insights for the evaluation of heat risk. The manuscript would be suitable for publication with minor clarifications and enhancements to its presentation.

Author’s Response:

We break the whole comments into points to provide a clear answer to each of them. The point-by-point responses are given below.

1. Clarification of the modelling contribution given that the Heat Index is deterministic.

Comment:

The Heat Index is derived from temperature and relative humidity, and as such, the manuscript should provide a more detailed explanation of the modelling approach's contribution.

Response:

We thank the reviewer for this important observation. To clarify the contribution of the proposed framework, we have explicitly emphasized throughout the manuscript (Abstract, Introduction, and Discussion) that the Heat Index (HI) is a deterministic function of air temperature and relative humidity. Accordingly, our framework does not aim to independently forecast HI, but instead performs conditional estimation based on meteorological predictors.

We have further strengthened this clarification by adding explicit statements highlighting that the key contribution of this study lies in capturing the nonlinear temporal dynamics, persistence, and high-frequency variability of HI using machine-learning approaches. This enables the transformation of meteorological inputs into operationally relevant, sub-daily heat-risk information, which is not directly obtainable from the static HI formulation. These revisions clearly distinguish the methodological contribution from the deterministic nature of HI and prevent misinterpretation as an independent forecasting system.

2. Ensuring conclusions are formulated with appropriate caution.

Comment:

It should guarantee that conclusions are formulated with care.

Response:

We have carefully revised the manuscript to ensure that all conclusions are stated with appropriate caution. Specifically, we consistently describe the results as conditional estimates and scenario-based projections, rather than deterministic forecasts. We also explicitly state that the proposed framework should complement rather than replace traditional meteorological forecasting systems, and that the projections depend on assumed meteorological inputs. These clarifications have been incorporated throughout the Abstract, Discussion, and Conclusion sections.

3. Additional detail on model validation, forecasting horizon, and uncertainty.

Comment:

Furthermore, providing more detailed information regarding model validation, forecasting horizons, and uncertainty in projections would enhance methodological transparency.

Response:

We thank the reviewer for this important suggestion. We have enhanced methodological transparency by adding explicit clarification statements within the relevant sections to better articulate the modeling framework.

� Forecasting horizon: We clarified that the framework operates as a 3-hour-ahead recursive conditional estimation system, and explicitly stated that longer-term outputs (2024–2027) represent scenario-based projections rather than direct long-horizon forecasts.

� Scenario interpretation: We added statements emphasizing that projections should not be interpreted as deterministic climate forecasts, but rather as conditional scenario analyses based on assumed meteorological inputs.

� Uncertainty quantification: We further clarified that the empirical tree-based prediction intervals reflect conditional model uncertainty under fixed inputs and do not capture the full uncertainty associated with future climate variability or extreme events.

� Model validation: We added a clarifying statement highlighting that the validation procedures (including expanding-window cross-validation and residual diagnostics) ensure robust generalization under temporally ordered data while minimizing overfitting and information leakage.

These additions improve clarity without altering the underlying methodology.

4. Minor language polishing

Comment:

Minor language polishing would also enhance the readability of certain sections.

Response:

We have carefully reviewed the manuscript and performed minor language refinements to improve clarity and readability, including simplifying long sentences and improving phrasing where necessary.

Attachment Submitted filename: Response_to_Reviewer_05.pdf

# Peer review: Decision Letter 5

**DOI:** 10.1371/journal.pone.0348202.r011

**Authors:** Lingye Yao

13 Apr 2026

Machine-Learning Framework for Conditional Estimation and Scenario-Based Projection of the Heat Index for Public Health Interventions

PONE-D-25-44349R5

Dear Dr. Binte Ahmed,

We’re pleased to inform you that your manuscript has been judged scientifically suitable for publication and will be formally accepted for publication once it meets all outstanding technical requirements.

Within one week, you’ll receive an e-mail detailing the required amendments. When these have been addressed, you’ll receive a formal acceptance letter and your manuscript will be scheduled for publication.

An invoice will be generated when your article is formally accepted. Please note, if your institution has a publishing partnership with PLOS and your article meets the relevant criteria, all or part of your publication costs will be covered. Please make sure your user information is up-to-date by logging into Editorial Manager at Editorial Manager® and clicking the ‘Update My Information' link at the top of the page. For questions related to billing, please contact  and clicking the ‘Update My Information' link at the top of the page. For questions related to billing, please contact  and clicking the ‘Update My Information' link at the top of the page. For questions related to billing, please contact  and clicking the ‘Update My Information' link at the top of the page. For questions related to billing, please contact billing support....

If your institution or institutions have a press office, please notify them about your upcoming paper to help maximize its impact. If they’ll be preparing press materials, please inform our press team as soon as possible -- no later than 48 hours after receiving the formal acceptance. Your manuscript will remain under strict press embargo until 2 pm Eastern Time on the date of publication. For more information, please contact onepress@plos.org.

Kind regards,

Lingye Yao, Ph.D.

Academic Editor

PLOS One

Additional Editor Comments (optional):

Please ensure to make Minor Revision raised by Reviewer#1 at the proofreading stage.

Reviewers' comments:

Reviewer's Responses to Questions

**Comments to the Author**

Reviewer #1: All comments have been addressed

**********

2. Is the manuscript technically sound, and do the data support the conclusions?

Reviewer #1: Yes

**********

3. Has the statistical analysis been performed appropriately and rigorously?

Reviewer #1: Yes

**********

4. Have the authors made all data underlying the findings in their manuscript fully available?

The PLOS Data policy requires authors to make all data underlying the findings described in their manuscript fully available without restriction, with rare exception (please refer to the Data Availability Statement in the manuscript PDF file). The data should be provided as part of the manuscript or its supporting information, or deposited to a public repository. For example, in addition to summary statistics, the data points behind means, medians and variance measures should be available. If there are restrictions on publicly sharing data—e.g. participant privacy or use of data from a third party—those must be specified.requires authors to make all data underlying the findings described in their manuscript fully available without restriction, with rare exception (please refer to the Data Availability Statement in the manuscript PDF file). The data should be provided as part of the manuscript or its supporting information, or deposited to a public repository. For example, in addition to summary statistics, the data points behind means, medians and variance measures should be available. If there are restrictions on publicly sharing data—e.g. participant privacy or use of data from a third party—those must be specified.

Reviewer #1: Yes

**********

5. Is the manuscript presented in an intelligible fashion and written in standard English?

Reviewer #1: Yes

**********

Reviewer #1: The revised manuscript presents a clear, well-structured analysis of Heat Index estimation and scenario-based projection using machine learning, addressing an important issue of urban heat stress and public health in Dhaka. The authors have substantially improved the manuscript by clarifying the conceptual framework, particularly the distinction between deterministic Heat Index formulation and conditional estimation, as well as differentiating short-term prediction from scenario-based projections. The methodological rigour has been strengthened through appropriate time-series cross-validation, residual diagnostics, and the inclusion of uncertainty estimation via empirical prediction intervals. The manuscript is now technically sound, and the results are generally well supported by the data. The conclusions are appropriately framed with necessary caution, avoiding overstatement. Minor improvements could still be made by further enhancing the clarity of model contribution and ensuring complete transparency in data and code availability to fully align with open science practices. Overall, the study provides useful insights for heat-risk assessment and is suitable for publication after minor final refinements.

**********

what does this mean?). If published, this will include your full peer review and any attached files.). If published, this will include your full peer review and any attached files.). If published, this will include your full peer review and any attached files.). If published, this will include your full peer review and any attached files.

If you choose “no”, your identity will remain anonymous but your review may still be made public.

**Do you want your identity to be public for this peer review?** For information about this choice, including consent withdrawal, please see our For information about this choice, including consent withdrawal, please see our For information about this choice, including consent withdrawal, please see our For information about this choice, including consent withdrawal, please see our Privacy Policy..

Reviewer #1: **Yes:** SAYON PRAMANIKSAYON PRAMANIKSAYON PRAMANIKSAYON PRAMANIK

**********

# Editor report: Acceptance letter

**DOI:** 10.1371/journal.pone.0348202.r012

**Authors:** Lingye Yao

PONE-D-25-44349R5

PLOS One

Dear Dr. Binte Ahmed,

I'm pleased to inform you that your manuscript has been deemed suitable for publication in PLOS One. Congratulations! Your manuscript is now being handed over to our production team.

At this stage, our production department will prepare your paper for publication. This includes ensuring the following:

* All references, tables, and figures are properly cited

* All relevant supporting information is included in the manuscript submission,

* There are no issues that prevent the paper from being properly typeset

You will receive further instructions from the production team, including instructions on how to review your proof when it is ready. Please keep in mind that we are working through a large volume of accepted articles, so please give us a few days to review your paper and let you know the next and final steps.

Lastly, if your institution or institutions have a press office, please let them know about your upcoming paper now to help maximize its impact. If they'll be preparing press materials, please inform our press team within the next 48 hours. Your manuscript will remain under strict press embargo until 2 pm Eastern Time on the date of publication. For more information, please contact onepress@plos.org.

You will receive an invoice from PLOS for your publication fee after your manuscript has reached the completed accept phase. If you receive an email requesting payment before acceptance or for any other service, this may be a phishing scheme. Learn how to identify phishing emails and protect your accounts at https://explore.plos.org/phishing.

If we can help with anything else, please email us at customercare@plos.org.

Thank you for submitting your work to PLOS ONE and supporting open access.

Kind regards,

PLOS ONE Editorial Office Staff

on behalf of

Dr. Lingye Yao

Academic Editor

PLOS One
